# Next-generation sequencing analyses of the emergence and maintenance of mutations in CTL epitopes in HIV controllers with differential viremia control

**DOI:** 10.1186/s12977-018-0444-z

**Published:** 2018-09-10

**Authors:** Diogo Gama Caetano, Fernanda Heloise Côrtes, Gonzalo Bello, Sylvia Lopes Maia Teixeira, Brenda Hoagland, Beatriz Grinsztejn, Valdilea Gonçalves Veloso, Monick Lindenmeyer Guimarães, Mariza Gonçalves Morgado

**Affiliations:** 1 Laboratório de Aids e Imunologia Molecular, Instituto Oswaldo Cruz –FIOCRUZ, Av. Brasil 4365, Rio de Janeiro, RJ 21045-900 Brazil; 2 Laboratório de Pesquisa Clínica em DST e Aids, Instituto Nacional de Infectologia Evandro Chagas (INI)—FIOCRUZ, Rio de Janeiro, Brazil

**Keywords:** HIV-1, HIV controller, CTL epitope, Single-nucleotide polymorphism, Escape mutant, Next-generation sequencing

## Abstract

**Background:**

Despite the low level of viral replication in HIV controllers (HICs), studies have reported viral mutations related to escape from cytotoxic T-lymphocyte (CTL) response in HIV-1 plasma sequences. Thus, evaluating the dynamics of the emergence of CTL-escape mutants in HICs reservoirs is important for understanding viremia control. To analyze the HIV-1 mutational profile and dynamics of CTL-escape mutants in HICs, we selected 11 long-term non-progressor individuals and divided them into the following groups: (1) viremic controllers (VCs; n = 5) and (2) elite controllers (ECs; n = 6). For each individual, we used HIV-1 proviral DNA from PBMCs related to earliest (V_E_) and latest (V_L_) visits to obtain *gag* and *nef* sequences using the Illumina HiSeq system. The consensus of each mapped gene was used to assess viral divergence, and next-generation sequencing data were employed to identify SNPs and variations within and flanking CTL epitopes.

**Results:**

Divergence analysis showed higher values for *nef* compared to *gag* among the HICs. EC and VC groups showed similar divergence rates for both genes. Analysis of the number of SNPs showed that VCs present more variability in both genes. Synonymous/non-synonymous mutation ratios were < 1 for *gag* among ECs and for *nef* among ECs and VCs, exhibiting a predominance of non-synonymous mutations. Such mutations were observed in regions encoding CTL-restricted epitopes in all individuals. All ECs presented non-synonymous mutations in CTL epitopes but generally at low frequency (< 1%); all VCs showed a high number of mutations, with significant frequency changes between V_E_ and V_L_ visits. A higher frequency of internal mutations was observed for *gag* epitopes, with significant changes across visits compared to *Nef* epitopes, indicating a pattern associated with differential genetic pressure.

**Conclusions:**

The high genetic conservation of HIV-1 *gag* and *nef* among ECs indicates that the higher level of viremia control restricts the evolution of both genes. Although viral replication levels in HICs are low or undetectable, all individuals exhibited CTL epitope mutations in *proviral gag* and *nef* variants, indicating that potential CTL escape mutants are present in HIC reservoirs and that situations leading to a disequilibrium of the host-virus relationship can result in the spread of CTL-escape variants.

**Electronic supplementary material:**

The online version of this article (10.1186/s12977-018-0444-z) contains supplementary material, which is available to authorized users.

## Background

One of the main characteristics of HIV-1 infection is the occurrence of clinical, but not virological, latency between the acute and AIDS phases over time, with variable duration among infected individuals that generates distinct progression profiles. Although the majority of individuals present high viral loads during the chronic infection phase, evolving to AIDS after 8–10 years of infection, a small fraction remains clinically asymptomatic for a long period. These individuals have normal CD4^+^ T cell counts in the absence of antiretroviral treatment (ART) and are termed long-term non-progressors (LTNPs) [1]. Moreover, some individuals, called HIV controllers (HICs), exhibit spontaneous control of viral replication at different levels, maintaining low or undetectable viremia during infection [2].

A crucial characteristic of HIV-1 is the high genetic variability and elevated rate of intra-host viral evolution [3]. This higher rate of viral evolution favors the emergence of viral variants that are more cytopathic [4–6], resistant to ART [7] and/or constitute escape variants from the host immune response [8–11]. In addition to variants that show escape from neutralizing antibodies [12], CTL-escape mutations are important to HIV-1 pathogenesis, as much evidence points to the pivotal role of the CD8^+^ T cell response in viral control [13–16] and as a continuous force driving viral selection [17]. These escape mutations have been characterized as amino acid changes occurring in central (impairing TCR recognition) and terminal (modifying anchor residues) regions of CTL epitopes or in their flanking regions, impairing epitope processing [18]. CTL-escape mutations begin to arise during the acute phase of infection [19–22] and can be identified even at human population levels based on HLA profiles [23–25]. Especially in individuals with protective HLA alleles, like HLA B*57 and B*27, those mutations arise faster in higher numbers [26] and preferentially, at anchor residues or multisite [27].

Although different studies have identified low HIV-1 evolutionary rates in LTNPs and HICs [28–30], immune-escape variants continue to arise in patients with these profiles, mainly when harboring protective HLA-B alleles [17, 31, 32]. Moreover, HICs present an efficient CD8^+^ T cell response [33, 34] that can favor high selective pressure and the emergence of immune-escape variants [35]. In HICs, this phenomenon can generate new effective CD8^+^ T cell responses after viral escape and maintain viremia control [36, 37] or can result in a loss of viremia control and disease progression [38–40]. Regardless, studies have rarely detected CTL-escape mutants in proviral sequences from HICs, even when emerging in plasma viral sequences.

Next-generation sequencing (NGS) is shown a useful tool for studying the dynamics of minority HIV-1 variations in infected individuals. NGS has been successfully applied to reveal hidden mutations conferring resistance to antiretroviral drugs in treated patients [41–43], to monitor viral tropism change dynamics [43, 44], and to assess the dynamics of immune-escape mutations in HIV-1-infected individuals [16, 19, 20]. However, this approach has not yet been employed to evaluate CTL-escape mutations in HICs.

Thus, the present study aimed to apply the NGS strategy to evaluate the overall genetic variability of *gag* and *nef* genes and to identify the emergence of potential CTL-escape mutations in proviral DNA sequences from 11 LTNP/HICs during long-term follow-up.

## Methods

### Study subjects

A cohort of 11 HICs with an LTNP profile, defined as subjects infected with HIV-1 for at least eight years and maintaining RNA viral loads lower than 2000 copies/ml and CD4^+^ T cells counts higher than 500 cells/mm^3^ without ART, were followed-up at the Instituto Nacional de Infectologia Evandro Chagas (INI), Rio de Janeiro, Brazil. These subjects were classified into the following groups according to plasma viral load (VL): (1) elite controllers (ECs) if most (≥ 70%) plasma viral load determinations were below the limit of detection for clinically available assays (< 50 or < 80 copies/ml) (n = 6) and (2) viremic controllers (VCs) if most (≥ 70%) VL determinations were between 80 and 2000 copies/ml (n = 5). Patients were seen at least once every 6-12 months to perform clinical monitoring tests, such as RNA viral load quantification and CD4^+^ T cell count. At each visit, PBMCs were obtained as previously described [45] and stored in liquid nitrogen until use. The present work was approved by the Brazilian National Committee for Research Ethics, and all patients provided written informed consent.

### CD4^+^ T cell counts and plasma HIV-1 RNA quantification

Absolute CD4^+^ T cell counts were obtained using the MultiTest TruCount-kit and MultiSet software with a FACSCalibur flow cytometer (BD Biosciences, California, USA). Plasma VL was measured using the Nuclisens HIV-1 RNA QT assay (Organon Teknika, North Carolina, USA; limit of detection: 80 copies/ml) from 1999 to 2008, the Versant HIV-1 3.0 RNA assay (bDNA 3.0, Siemens, New York, USA; limit of detection: 50 copies/mL) from 2008 to 2013, and the Abbott RealTime HIV-1 assay (Abbott Laboratories, Wiesbaden, Germany; limit of detection: 40 copies/mL) from 2013 to 2016.

### Genomic DNA extraction and PCR

For each patient, PBMCs samples from the earliest (V_E_) and latest (V_L_) visits were used for DNA extraction. Thawed PBMCs (≅ 1 × 10^7^ cells for VCs and ≅ 2 × 10^7^ cells for ECs) were suspended in 1 ml of DNAZOL (Invitrogen, Wisconsin, USA) and incubated for 72 h at 4 °C. Genomic DNA was further extracted as previously described [46] and used to amplify fragments related to regions 408-1844 and 8697-9639 of the HIV-1 Genome (in relation to HXB2), encompassing *gag* and *nef,* respectively, as described elsewhere [45]. The primer sets are described in Additional file 1: Table S1. The PCR reactions were carried out by using Platinum^®^ Taq DNA Polymerase High Fidelity (Invitrogen) according to the manufacturer’s protocol. To avoid using samples near or at the limit of detection, all samples were previously tested in triplicate via nested PCR, and only samples with at least 2 positive reactions of 3 total were included in the study (data not shown). The PCR products were purified using an Illustra GFX PCR DNA purification kit (GE Healthcare, Pennsylvania, USA) and quantified with Qubit dsDNA BR Assay Kit (Invitrogen) using a Qubit^®^ 2.0 fluorometer (Invitrogen).

### Library preparation and NGS

Purified *gag* and *nef* amplicons obtained for each patient visit were multiplexed in equimolar pools and used to construct an NGS genomic library with Nextera XT DNA Library kits (Illumina, California, USA) and Nextera XT Index Kit (Illumina), according to the manufacturer’s instructions. The generated libraries were normalized and clustered using HiSeq SR Rapid Cluster V2 (Illumina). NGS was performed using HiSeq Rapid SBS v2 of 200 cycles (Illumina) with an Illumina HISEQ 2500 sequencer (Illumina).

### Data analysis

The raw NGS data obtained were compiled into FastQ files, and the quality of the reads was assessed by using the software FastQC [47]. The tool Trimmomatic v0.32 [48] was used to trim adapter sequences, and the first 100 bp of the reads were obtained. Furthermore, Sickle [49] was employed to select only sequences with size > 150 bp and Q > 30. Reads were mapped separately to *gag* and *nef* sequences from HXB2 (GenBank accession K03455) with Geneious 9.0.5 [50] by using medium sensitivity and 5 iterations with the Geneious mapping algorithm. Consensus sequences with a 90% base threshold and a minimum coverage of 20X for each mapping were obtained through Geneious. Reads were then remapped using obtained consensus sequences as a reference to filter differences between HXB2 and individual HIV-1 quasispecies. Samtools [51] was utilized to obtain mapping coverage statistics. Genetic divergence calculations between the consensus for V_E_ and V_L_ and Neighbor-Joining (NJ) trees with 1000 bootstrap replicates were generated with Mega v.6 and by using the Tamura Nei substitution model, as recommended by jModel test [52]. For each mapping, variant call analysis was performed in Geneious software to assess single-nucleotide polymorphisms (SNPs); variation at Q > 30, coverage > 100X and frequency > 0.5% were used as NGS sequencing quality parameters. Relevant CTL epitopes, restricted by the HLA-B alleles carried by the individuals included in the study group, were selected from the epitope database of Los Alamos HIV Immunology Database [53] available September 2017 (Additional file 1: Table S2 and S3) and used to identify variations within or adjacent (3 amino acids flanking the sequence) to epitopes that may be related to the immune response.

### Statistical analysis

GraphPad Prism 6 was used to plot graphs and to estimate the median values of divergence per year, number of variants, ratio of variable positions/total of positions and synonymous/non-synonymous mutation ratio. The Mann–Whitney U test was performed using R v3.4 to compare *gag* and *nef* genes from the HIV-1 EC and VC groups. p values < 0.05 were considered statistically significant.

## Results

### Clinical and demographic characteristics of the cohort

Table 1 describes the clinical and epidemiologic characteristics of a group of HIV-infected individuals from the INI cohort classified as LTNPs and HICs. The median age of the individuals was 48 years (IQR 45–51); most of them (67%) were women, and the heterosexual category of exposure was prevalent (58%). The median time of HIV-1 suppression was 15 years (IQR 14–18). The medians of CD4^+^ and CD8^+^ T cells counts were compatible with the values currently described for HIV-1-uninfected individuals [54]. Among the 11 individuals analyzed, all were infected with HIV-1 variants genotyped as subtype B, except for VC14, who was infected with subtype F1. Six of 9 patients carried HLA-B allele B*57 or B*52, associated with slow clinical progression [55–57]. Two individuals were heterozygotes for CCR5Δ32 deletion, also described as a protective factor [56]. EC17, VC14, and EC42 did not carry an HLA-B allele or exhibit a CCR5 genetic profile associated with the control of viral replication and/or non-progression to AIDS.

**Table 1:** Clinical and epidemiological characteristics of individuals in the study

Patient	Gender	Age (years)	Year of HIV diagnosis	Years of HIV suppression	Exposure category	Median CD4^+^ T cells (cells/mm^3^)	Median CD8^+^ T cells (cells/mm^3^)	Viral load frequency (%)		
						(IQR)	(IQR)	< 80^a^	81–400^a^	401–5000^a^
EC02	Female	52	1997	15	HET	1229 (1088–1443)	1539 (1406–1741)	100		
EC17	Female	65	2000	15	NI	1771 (1505–2134)	836 (693–994)	90	10	
EC52	Female	43	1997	18	HET	1263 (1056–1420)	485 (423–540)	100		
EC11	Female	48	1995	20	HET	1078 (987–1218)	965 (806–1168)	92		8
EC18	Female	82	2001	9	HET	809 (675–940)	666 (594–761)	87	13	
EC42	Female	61	1993	22	HET	974.5 (871–1133)	734 (515–933)	74	26	
VC05	Male	51	1991	24	NI	1278 (1114–1461)	857 (645–1009)	16	47	17
VC06	Male	37	2000	11	MSM	1093 (929–1222)	1112 (870–1224)	42	50	8
VC14	Female	45	1999	16	HET	701.5 (652–767)	657 (585–749)	55	36	9
VC15	Female	41	2001	14	HET	703 (677–826)	894 (755–1002)			100
VC16	Male	48	1998	17	MSM	563 (528–637)	837 (719–958)	21	37	32

Patient	Genotype HLA-B	Genotype CCR5	Early visit date	Later visit date	Difference V_E _× V_L_ (months)
EC02	B*48, B*52	WT/WT	NOV/08	AUG/12	45
EC17	B*07, B*40	WT/WT	SEP/09	OCT/13	49
EC52	B*45, B*57	WT/WT	FEB/09	AUG/13	54
EC11	B*49, B*81	*WT/∆32*	DEC/09	APR/12	28
EC18	B*07, B*52	WT/WT	OCT/09	SEP/10	11
EC42	B*15, B*51	WT/WT	DEC/09	NOV/14	59
VC05	B*15, B*52	WT/WT	FEB/09	JUN/13	52
VC06	B*15, B*48	*WT/∆32*	JAN/09	MAY/11	28
VC14	B*42, B*44	WT/WT	APR/09	NOV/14	67
VC15	B*56, B*57	WT/WT	AUG/09	FEB/13	42
VC16	B*14, B*57	WT/WT	SEP/09	SEP/14	60

^a^Copies/ml

*HET* heterosexual, *NI* non-Informed, *MSM* men who have sex with men, *IQR* interquartile range, *WT* wild-type, *HLA-B* protective alleles are in italic

### NGS data yield and gene mapping

From the NGS, a general median number of 4,667,122 reads (IQR 5,491,122–3,688,230) of 35–200 bp were obtained per visit from each individual. Approximately 70% of reads (IQR 68–76%) were retained after selection by size and quality controls. Additional file 1: Table S4 shows the mapping coverage for both genes of each patient per visit. For *gag*, the median coverage was 212,674 reads/bp (IQR 173,132–261,207) per individual/per visit. The data generated allowed reconstruction of the consensus sequence for at least the first 1050 bp of *gag*. However, *gag* mapping was not successful for EC17 and EC42 samples at the V_E_ visit. With regard to the *nef* gene, the median coverage was 286,463 reads/bp (IQR 179,882–426,834) per individual/per visit. The generated data allowed reconstruction of the consensus sequence of 821 bp, covering full-length *nef*. Correct *nef* mapping was not successful for VC06 V_E_ and EC17 V_L_ visit samples.

### Genetic diversity of *gag* and *nef* regions among HICs

**Fig. 1:**
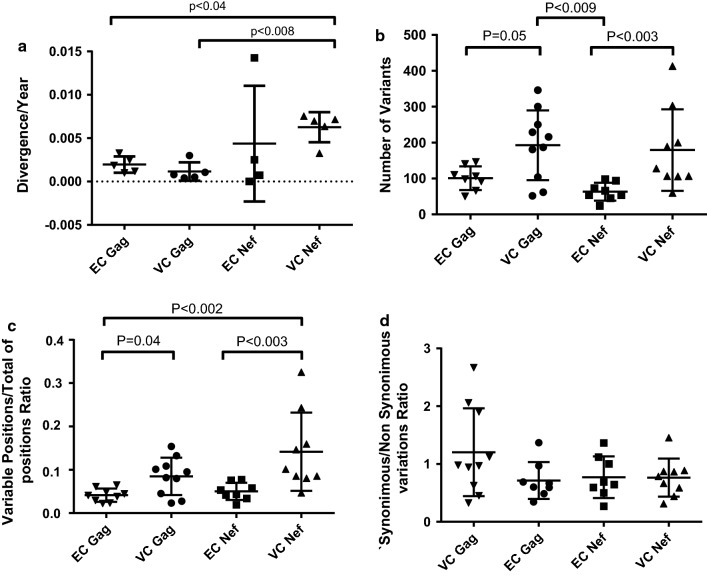
Divergence/year (**a**), number of variants (**b**), variable positions/total positions ratio (**c**) and synonymous/non-synonymous variation ratio (**d**) for *gag* and *nef* of HIV-1 infected VC and EC groups. Divergence was calculated as the genetic distance between V_E_ and V_L_ of each patient and normalized by year. Values obtained by NGS data for each visit and each patient for parameters (**b**–**d**) were plotted as individual values. Median values are indicated in the figure. The Mann–Whitney U test was used to compare groups. A p value < 0.05 was considered significant

To estimate evolution of the studied viral genes, we calculated the genetic divergence between the V_E_ and V_L_ consensus sequences obtained from HICs, normalizing the values by year according to the follow-up time, and compared them between ECs and VCs (Fig. 1a). For *nef* sample VC06 V_E_ and *gag* samples EC17V_E_/EC42 V_E_, which were not successfully mapped by NGS, bulk sequences available from previous studies using conventional Sanger sequencing [58] were employed. Among the HICs, the median of viral divergence per year was significantly higher for *nef* compared to *gag* (0.6 vs 0.1%; p < 0.03). Similar divergence rates were observed comparing EC and VC groups for both genes. Compared with *gag* from the VC (p < 0.008) and EC (p < 0.04) groups, *nef* from the VC group showed significantly higher divergence rates.

We used variant call analysis to quantify and qualify SNPs and variations identified in the NGS data for each individual and visit. For both genes, VCs showed a higher number of variations (Fig. 1b) than did ECs (p = 0.05 for *gag* and p < 0.003 for *nef*). Comparison of the variable position ratio per total analyzed positions showed the same pattern (Fig. 1c; *gag* VCs vs ECs p < 0.04; *nef* VCs vs ECs p < 0.03). Otherwise, *gag* in VCs had a higher number of variants than did *nef* in ECs (p < 0.009), whereas *nef* from VCs showed higher ratios of variable positions than did *gag* from ECs (p < 0.002). Synonymous/non-synonymous mutation ratios were not significantly different between the HIC groups or the studied genes, even though the median ratio for VC *gag* was greater than one and the ratios for *gag* in ECs and *nef* in both groups were less than one (Fig. 1d).

### Variability in CTL restricted epitopes in HICs

To assess the occurrence of potential Gag and Nef protein immune-escape mutations in the HICs included in this study, we identified for each individual the epitopes described in the literature as restricted by their HLA-B alleles. We further analyzed all non-synonymous SNPs associated with those epitope regions for each patient by comparing their frequencies between V_E_ and V_L_. Tables 2 and 3 show HICs carrying epitope mutations with a frequency change > 10% between visits, as distributed according to the HLA-B allele. Full lists of Gag and Nef epitope mutations for all the individuals included in this study are available as Additional file 1: Tables S5 and S6.

**Table 2:** Gag mutations with significant frequency changes across V_E_ and V_L_ and their associated epitopes

Patient	Mutation	Frequency (%)		Epitope			
		V_E_	V_L_	HLA	Position	Sequence	Location
EC02	A83S	0.0	22.9	B52	Gag (74–82)	ELRSLYNTV	A
	S278C	0.0	22.8	B52	Gag (275–282)	RMYSPTSI	I
EC11	K26R	0.0	94.1	B81	Gag (19–27)	IRLRPGGKK	I
EC42	E40Q	E^a^	17.7	B51	Gag (36–44)	WASRELERF	I
	N126S	S^a^	79.9	B15	Gag (127–135)	QVSQNYPIV	A
	N126R	S^a^	21.0	B15	Gag (127–135)	QVSQNYPIV	A
EC18	K28Q	33.9	0.0	B07	Gag (22–30)	RPGGKKHYM	I
	K28R	65.9	99.6	B07	Gag (22–30)	RPGGKKHYM	I
	I34L	21.0	99.8	B52	Gag (34–44)	LVWASRELERF	I
	R39K	0.0	99.5	B52	Gag (34–44)	LVWASRELERF	I
	R43Q	17.2	0.0	B52	Gag (34–44)	LVWASRELERF	I
	R76K	20.2	0.0	B07	Gag (71–79)	GSEELRSLY	I
	T280A	79.7	0.0	B07/B52	Gag (274–282)/Gag (275–282)	VRMYSPVSI/RMYSPTSI	I
	T280V	19.9	0.0	B07/B52	Gag (274–282)/Gag (275–282)	VRMYSPVSI/RMYSPTSI	I
	T280S	0.0	99.7	B07/B52	Gag (274–282)/Gag (275–282)	VRMYSPVSI/RMYSPTSI	I
VC10	N126S	0.7	94.2	B15	Gag (127–135)	QVSQNYPIV	A
	N126S	0.0	5.6	B15	Gag (127–135)	QVSQNYPIV	A
	A146P	39.9	37.6	B15	Gag (144–152)/Gag (147–155)	HQAISPRTL/ISPRTLNAW	I
	A146S	59.3	62.4	B15	Gag (144–152)/Gag (147–155)	HQAISPRTL/ISPRTLNAW	I
	S173T	43.1	67.5	B15	Gag (168–175)	VIPMFSAL	I
	I223V	41.6	32.4	B15	Gag (226–236)	GQMREPRGSDI	A
	T280S	45.3	66.7	B15/B52	Gag (274–282)/Gag (275–282)	VRMYSPTSI/RMYSPTSI	I
	T280I	52.7	17.4	B15/B52	Gag (274–282)/Gag (275–282)	VRMYSPTSI/RMYSPTSI	I
VC14	V82I	99.7	77.9	B44	Gag (78–86)	LYNTVATLY	I
	C87Y	97.4	99.7	B44	Gag (78–86)	LYNTVATLY	A
	I147L	99.7	81.1	B42	Gag (144–152)	HQAISPRTL	I
	S310T	99.7	81.1	B44	Gag (306–316)	AEQASQDVKNW	I
VC15	V82I	43.4	84.9	B57	Gag (76–86)	RSLYNTVATLY	I
	D121A	30.6	81.8	B57	Gag (114–122)	KTQQAAADK	I
	T122A	49.2	82.1	B57	Gag (114–122)	KTQQAAADK	I
	H124N	59.7	0.0	B57	Gag (114–122)	KTQQAAADK	A
	N271T	0.0	24.7	B57	Gag (274–282)	VRMYSPVSI	A
	T280V	1.8	98.8	B57	Gag (274–282)	VRMYSPVSI	I
VC16	I34L	32.2	0.0	B57	Gag (34–44)	LVWASRELERF	I
	V35I	32.6	0.0	B57	Gag (34–44)	LVWASRELERF	I
	V46I	29.3	0.0	B57	Gag (34–44)	LVWASRELERF	A
	V82L	25.9	0.0	B57	Gag (76–86)	RSLYNTVATLY	I
	A118P	24.9	0.0	B57	Gag (114–122)	KTQQAAADK	I
	A119T	24.9	0.0	B57	Gag (114–122)	KTQQAAADK	I
	T122A	4.8	71.6	B57	Gag (114–122)	KTQQAAADK	I
	G123K	24.7	0.0	B57	Gag (114–122)	KTQQAAADK	A
	H124N	99.1	64.4	B57/B14	Gag (114–122)/Gag (127–135)	KTQQAAADK/QVSQNYPIV	A
	H124S	0.7	35.3	B57/B14	Gag (114–122)/Gag (127–135)	KTQQAAADK/QVSQNYPIV	A
	S125R	30.9	0.0	B57/B14	Gag (114–122)/Gag (127–135)	KTQQAAADK/QVSQNYPIV	A
	Q127H	76.7	98.3	B14	Gag (127–135)	QVSQNYPIV	I
	I138L	75.5	94.1	B14	Gag (127–135)	QVSQNYPIV	A
	A146P	74.6	99.4	B57	Gag (145–155)	QAISPRTLNAW	I
	A163G	78.3	75.4	B14/B57	Gag (160–168)/Gag (162–172)	EEKAFSPEV/KAFSPEVIPMF	I
	S165N	77.6	75.0	B14/B57	Gag (160–168)/Gag (162–172)	EEKAFSPEV/KAFSPEVIPMF	I
	V168T	0.0	22.8	B14/B57	Gag (160–168)/Gag (162–172)	EEKAFSPEV/KAFSPEVIPMF	I
	S173T	37.7	74.1	B57	Gag (162–172)	KAFSPEVIPMF	A
	V191I	33.3	99.4	B14	Gag (183–191)	DLNMMLNIV	I
	T242N	78.4	99.5	B57	Gag (240–249)	TSTLQEQIGW	I
	D295N	0.0	9.9	B14	Gag (298–306)	DRFFKTLRA	A
	K335R	49.2	58.9	B14	Gag (329–337)	DCKTILKAL	I
	A340G	70.9	96.6	B14	Gag (329–337)	DCKTILKAL	A

^a^Consensus amino acid from the available bulk sequence shown instead of frequency; Location A—adjacent to epitope; Location I—within epitope

**Table 3:** Nef mutations with significant frequency changes across V_E_ and V_L_ and their associated epitopes

Patient	Mutation	Frequency (%)		Epitope			
		V_E_	V_L_	HLA	Position	Sequence	Location
EC11	V133P	70.7	99.4	B49	Nef (136–145)	PLTFGWCYKL	A
	F191 V	85.5	60.2	B81	Nef (183–192)	WRFDSRLAFH	I
EC42	V10R	99.0	8.4	B15	Nef (13–20)	WPAIRERM	A
	V10 K	0.0	91.0	B15	Nef (13–20)	WPAIRERM	A
	G12R	0.0	90.4	B15	Nef (13–20)	WPAIRERM	A
	M79I	0.0	89.6	B51/B15	Nef (72–81)/Nef (75–82)	PQVPLRPMTY/PLRPMTYK	I
	N126S	99.2	0.0	B51	Nef (120–128)	YFPDWQNYT	I
	Y135F	99.2	10.4	B15	Nef (137–145)	LTFGWCFKL	A
	G140R	0.0	88.5	B15	Nef (137–145)	LTFGWCFKL	I
	V148I	99.2	0.7	B15	Nef (137–145)	LTFGWCFKL	A
EC18	K92R	0.0	68.5	B07	Nef (83–91)/Nef (90–97)	AAVDLSHFL/FLKEKGGL	A/I
VC06	V85I	V^a^	45.2	B15	Nef (84–92)	AVDLSHFLK	I
	V85L	V^a^	28.3	B15	Nef (84–92)	AVDLSHFLK	I
	L87I	L^a^	56.9	B15	Nef (84–92)	AVDLSHFLK	I
	R105 K	R^a^	74.7	B15	Nef (106–114)	RQDILDLWI	A
	I114 V	I^a^	60.3	B15	Nef (106–114)/Nef (116–124)	RQDILDLWI/HTQGYFPDW	I/A
VC10	T15A	59.2	98.5	B15	Nef (13–20)	WPTVRERM	I
	E182Q	99.9	17.8	B15	Nef (183–191)	WRFDSRLAF	A
	E182L	0.0	81.8	B15	Nef (183–191)	WRFDSRLAF	A
	R188G	99.8	90.9	B15/B52	Nef (183–191)/Nef (188–196)	WRFDSRLAF/RLAFHHVAR	I
VC14	T71R	94.5	3.3	B42	Nef (71–79)	RPQVPLRPM	I
	I101 V	5.2	96.8	B44	Nef (92–100)	KEKGGLEGL	A
	H102Y	4.9	97.1	B44	Nef (92–100)/Nef (105–115)	KEKGGLEGL/KRQEILDLWVY	A
	H102 N	2.0	0.0	B44	Nef (92–100)/Nef (105–115)	KEKGGLEGL/KRQEILDLWVY	A
	H116 N	94.5	2.4	B44	Nef (105–115)	KRQEILDLWVY	A
	P129Q	5.7	96.5	B42	Nef (128–137)	TPGPGVRYPL	I
	V133I	5.2	96.5	B42	Nef (128–137)	TPGPGVRYPL	I
VC15	V85L	66.8	99.5	B57	Nef (82–90)	KAAFDLSFF	I
	H102Y	68.4	99.7	B57	Nef (105–115)	KRQEILDLWVY	A
VC16	Y81F	54.3	47.6	B57	Nef (82–90)	KAAFDLSFF	A
	H89Y	0.0	29.3	B57	Nef (82–90)/Nef (90–97)	KAAFDLSFF/FLKEKGGL	I/A
	H102Y	99.9	48.6	B14/B57	Nef (105–113)/Nef (105–115)	QRQDILDLW/KRQEILDLWVY	A
	H116 N	63.4	63.1	B57	Nef (105–115)/Nef (116–124)	KRQEILDLWVY/HTQGYFPDW	A/I
	V133T	99.4	13.3	B57	Nef (127–135)	YTPGPGIRY	I
	V133I	0.0	74.3	B57	Nef (127–135)	YTPGPGIRY	I

^a^Consensus amino acid from the available bulk sequence shown instead of frequency; Location A—adjacent to epitope; Location I—within epitope

Among HICs, EC individuals presented two main patterns of epitope mutation changes. For those individuals with none (EC02, EC52) or very few (EC17) viral blips during clinical follow-up, the majority of the epitope mutations detected were rare (< 2%) in both Gag and Nef, except for Gag epitope positions A83S (adjacent to EV9) and S278C (RI9), which each corresponded roughly to 23% in EC02 V_L_.

In addition, we observed mutations with major frequency changes between visits for all ECs who had more frequent viral blips (< 30%) during clinical follow-up (Tables 2 and 3). For EC11, we detected main changes in the frequency of Nef epitope mutations V133P (from 70.7 to 99.4%) and F191 V (from 85.5 to 60.2%) and Gag epitope mutation K26R (from 0 to 94.1%) between V_E_ and V_L_. For EC18, Nef epitope mutation K92R appeared only at V_L_ (68%); for Gag, epitope mutations K28R, I34L, R39 K and T280S became the majority at V_L_, whereas R43Q and R76K mutations decreased from V_E_ (approximately 20%) to undetectable levels at V_L_. EC13 showed the highest number of Nef changes in mutation frequency, such as SNPs V10 K, G12R, M79I, and G140R, which became predominant at V_L_; in contrast, reversion to subtype B consensus Y and V residues were observed for Y135F and V148I. For Gag, NGS data for EC13 at V_L_ revealed E40Q and N126R mutations in approximately 20%, but with no significant changes in comparison to the corresponding bulk sequence available for this individual.

As expected, VCs had more mutations with frequencies above 1% than did ECs. In addition to the major frequency changes, some patients also showed a high number of mutations with equivalent frequency throughout the visits. VC06 presented dominant changes in Nef mutations V85I, L87I, R105K, and I114V, despite no significant change in Gag. VC10 also presented major changes in Nef mutations T15A and E182L; for Gag, main alterations were observed in N126S and in co-circulation of A146P/A146S, T280S/T280I, S173T/S173, and I223V/I223 variants at both visits. VC14 showed reversion of Nef T71R and H116N and the emergence of I101V, H102Y, P129Q, and V133I at V_L_; for Gag, major changes were approximately 20% of the reversions for V82I, I147L, and S310T. Furthermore, VC15 presented main changes in Gag for V82I, D121A, T122A, and T280V, with reversion observed for H124N, whereas Nef V85L and H102Y increased by approximately 30%. VC16 presented a large number of amino acid changes, mainly in Gag epitopes, with reversion of approximately 30% for I34L, V35I, V46I, V82L, A118P, A119T, G123 K, and S125R mutations and an increase from 20 to 30% for H124S, Q1227H, I138L, A146P, V168T, T242 N and A340G from V_E_ to V_L_. Major changes from approximately 40–70% were also found for T122A, S173T, and V191I. Co-circulation of subtype B consensus amino acids with mutations was found for K335R/K335. For Nef, we observed co-circulation of wild-type and Y81F and H116N mutations, reversion of H102Y and major frequency changes of V133I (undetectable vs 74%) from V_E_ to V_L_.

## Discussion

HICs are a rare population of HIV-1-infected individuals who represent the best existing model of spontaneous viral control [2, 59, 60]. Although the mechanisms responsible for this control are not fully understood, studies show that these individuals have a differentiated and more effective CTL response [61], which should be then reflected in greater genetic pressure on their viral quasispecies, mainly in immunodominant regions. Moreover, the presence of protective HLA alleles related to clinical non-progression and/or viremia control is also associated with stronger selective pressure for virus diversity [17, 22, 26, 27, 31, 32, 35, 36, 62–66].

Although most HICs have plasma viral loads below the limit of detection of commercial assays, basal viral replication levels can be detected by ultrasensitive methods [59, 67, 68] and should favor viral evolution to some degree due to the characteristic high-genetic variability from HIV. Several studies have reported lower levels of viral diversity in HICs compared with the levels in typical progressors [28, 29, 32, 59, 69]. Gijsberg et al. estimated divergence rates of 0.9–1.9% over ten months for *gag* in HIV-1 samples from typical progressors [70]. In our study, a median of viral divergence of 0.1% per year was observed for *gag* gene from HICs, corroborating the low level of viral evolution found in HIV-1 samples from those individuals. A higher median of divergence was observed for *nef* (0.6% per year), suggesting that *nef* has a greater potential for viral diversity than does *gag,* in agreement with previous studies showing greater conservation of *gag* [71, 72]. Previous observations from our group of lower quasispecies diversity for the *env* gene from EC samples in comparison to VCs [73] and the low, but similar, median values of divergence in *gag* and *nef* for viral samples from ECs and VCs in the present study indicate that lower levels of viral replication restrict evolution.

To our knowledge, this is the first study employing NGS to analyze HIV-1 diversity in major CTL epitopes of HICs. Indeed, most similar studies were performed with SIV-infected primates with a viremia control profile [74, 75]. The use of the NGS platform allowed the estimation of variability in terms of SNP quantity and nature. Previously, Cale et al. [76] using 454 sequencing, showed that full coverage of 50,000 reads/bp was sufficient to detect variants with frequencies of 0.006%. In our work, we used a medium coverage of > 200,000 reads/bp to assess SNPs with frequencies higher than 0.5%, which should identify the most representative escape mutation variants while preventing analysis of data related to sequencing artifacts. With this approach, we showed higher levels of variants and variable positions in the *gag* and *nef* genes of viral samples from VCs compared to ECs, correlating with the higher variability expected for the first group. Although differences between the synonymous/non-synonymous mutation ratios of both groups and genes were not statistically significant, *gag* of ECs and *nef* of ECs and VCs displayed a predominance of non-synonymous mutations, in contrast with previous studies reporting that synonymous mutations are more significant to evolution of *gag* and *nef* [32, 77].

To characterize possible CTL-escape mutants, we performed analysis of variations in the epitopes restricted by each patient’s HLA-B allele. Similar molecular analysis has been able to identify that most mutations arising in the first weeks of the acute phase are the result of CTL response selective pressure [13, 19–22]. Concerning HICs, Migueles et al. [78] showed a low frequency of CTL-escape mutations for the KF11 epitope, despite its high level of CTL recognition in individuals carrying the B57 allele. Additionally, a more in-depth description of *gag* and *nef* gene evolution in B57^+^ elite suppressors showed that despite the predominance of immune-escape mutations in *gag* and *nef* quasispecies obtained from plasma viral RNA, these mutations are rare in proviral sequences [32, 65, 77, 79].

In our study, non-synonymous mutations were found in Gag and Nef CTL epitope regions in all HIV-controllers regardless of their rarity in the proviral compartment. Due to the low HIV proviral load inherent to ECs, a higher number of PBMCs was used for DNA extraction than for VCs (≅ 2 × 10^7^ cells for ECs vs ≅ 1 × 10^7^ cells for VCs) in order to assure a proviral input in the nested-PCR sufficient to assess the viral variability in each sample. Moreover, all samples were tested in triplicate and only those with at least 2 out of three positive nested-PCR amplification were used to prepare the NGS amplicons. These strategies were employed to prevent low input of viral copies on PCR that could lead to template resampling. Moreover, the high number of sequences generated from each sample and the higher sensitivity of NGS to access minority viral variants, in contrast to techniques such as single-genome amplification (SGA) or cloning [19, 20], allowed the detection of those mutations. The occurrence of unique low-frequency mutations for both early and late visit samples from the same individual showed that in contrast to the results of Bailey et al. [65], possible escape mutants from the CTL response replicating in the plasma compartment can successfully integrate into host cells. Although this low frequency might appear to be insignificant, new CTL-escape mutants do not often arise in massive frequencies but can expand from very low to predominant conditions, as previously exemplified for SIV [80] and observed in our work for the following mutations: Gag-N126S (0.7 –> 99.8%) for VC10 and Gag-T280V (1.8 –> 98.8%) for VC15.

Comparing Gag and Nef mutations with significant frequency changes revealed that mutations within the analyzed epitopes predominantly occurred in Gag, whereas no pattern of mutation was present in Nef epitopes, within or in adjacent regions. Although both types of mutations can generate CTL escape through different mechanisms, mutations within epitope are more easily associated with the escape profile, as it directly affects epitope anchoring and TCR recognition [18, 81]. This observation may be related to the predominance of the CTL response associated with Gag during the chronic phase of infection [13, 15, 22, 34], generating higher selective pressure in this gene and resulting in greater diversity of the epitopes.

In general, the low number of patients in each group is a limitation to identifying statistically significant associations between the level of viremia control and the emergence dynamics of mutations related to CTL epitopes. The low viral load observed for ECs was also a limitation to assessing escape mutations in the plasma compartment, which reflects the variants that are effectively replicating in the host. However, by evaluating the proviral reservoir, which represents the pool of viruses that can be a source of plasma viral particles, we were able to assess a greater number of mutations with significant frequency changes either in VCs or ECs. Although some ECs, such as EC08, did not present any significant change across visits, all VCs showed mutations that characterized variant replacement with regard to both Nef and Gag. In those patients, we were able to observe co-circulation of more than one mutation in the same position, which is indicative of greater dynamic quasispecies turnover. Reversion to wild-type amino acids, as based on the reference subtype, was also observed for VCs, and reversion of escape mutations has been extensively described in the literature as a common viral mechanism of evolution related to the CTL response [81–86].

For the individuals EC08, EC18, VC15 and VC16, who carried the protective HLA* B57 allele, analyses of IW9 (Gag 147-155), KF11 (Gag 162-172) and TW10 (Gag 240-249) epitopes indicated a low level of viral evolution, even in these HIV-1 CTL epitopes related to high selective pressure [22, 35, 36, 62–66]. Although EC08 and EC18 individuals presented wild-type amino acids at all positions of the epitopes analyzed, VC15 presented I147L, A146P, and T242 N mutations, and VC16 harbored A146P, A163G, and T242 N mutations. All of the mutations identified herein have been described in several studies as commonly arising in individuals carrying HLA-B*57 and B*58 alleles, despite resulting in a loss of viral fitness [18, 36, 65, 66].

## Conclusion

Although none of the observed mutations could be confirmed as a CTL-escape mutation due to the lack of CD8^+^ T cell functional analyses, the present study shows that despite low or undetectable levels of viral replication among HICs, genetic variability occurs in viral quasispecies in the proviral compartment. Amino acid substitutions across visits and the existence of low-frequency mutants, even in ECs, indicate that potential CTL-escape mutants exist and are present in those individual reservoirs. This fact implies that situations leading to a disequilibrium of the host-virus relationship can result in the spread of CTL-escape variants with pathological consequences. More studies are necessary to address why those adapted variants do not achieve replicative success in ECs.

## Additional file

**Additional file 1.Table S1.** Primer set used in the present study. **Table S2.** Gag Epitopes selected for study according to the HLA-B alleles carried by the HICs. **Table S3**. Nef Epitopes selected for study according to the HLA-B alleles carried by the HICs. **Table S4.** NGS mapping and coverage statistics of *gag* and *nef* distributed according to the patients. **Table S5.** Full list of Gag mutations and associated epitopes recognized by HLA alleles carried by the HICs. **Table S6.** Full list of Nef mutations and associated epitopes recognized by HLA alleles carried by the HICs

## References

[1] Pantaleo G, Fauci AS (1996). Immunopathogenesis of HIV infection. Annu Rev Microbiol.

[2] Sáez-Cirión A, Pancino G (2013). HIV controllers: a genetically determined or inducible phenotype?. Immunol Rev.

[3] Shankarappa R, Margolick JB, Gange SJ, Rodrigo AG, Upchurch D, Farzadegan H (1999). Consistent viral evolutionary changes associated with the progression of human immunodeficiency virus type 1 infection. J Virol.

[4] Tersmette M, Gruters RA, de Wolf F, de Goede RE, Lange JM, Schellekens PT (1989). Evidence for a role of virulent human immunodeficiency virus (HIV) variants in the pathogenesis of acquired immunodeficiency syndrome: studies on sequential HIV isolates. J Virol.

[5] Dalmau J, Rotger M, Erkizia I, Rauch A, Reche P, Pino M (2014). Highly pathogenic adapted HIV-1 strains limit host immunity and dictate rapid disease progression. AIDS.

[6] Hayashida T, Tsuchiya K, Kikuchi Y, Oka S, Gatanaga H (2017). Emergence of CXCR4-Tropic HIV-1 variants followed by rapid disease progression in hemophiliac slow progressors. PLoS ONE.

[7] Chen R, Quinones-Mateu ME, Mansky LM (2004). Drug resistance, virus fitness and HIV-1 mutagenesis. Curr Pharm Des.

[8] Koenig S, Conley AJ, Brewah YA, Jones GM, Leath S, Boots LJ (1995). Transfer of HIV-1-specific cytotoxic T lymphocytes to an AIDS patient leads to selection for mutant HIV variants and subsequent disease progression. Nat Med.

[9] Erdmann N, Du VY, Carlson J, Schaefer M, Jureka A, Sterrett S (2015). HLA class-II associated HIV polymorphisms predict escape from CD4 + T cell responses. PLoS Pathog.

[10] Dalmau J, Rotger M, Erkizia I, Rauch A, Reche P, Pino M (2014). Highly pathogenic adapted HIV-1 strains limit host immunity and dictate rapid disease progression. Aids.

[11] Ouyang Y, Yin Q, Li W, Li Z, Kong D, Wu Y (2017). Escape from humoral immunity is associated with treatment failure in HIV-1-infected patients receiving long-term antiretroviral therapy. Sci Rep.

[12] Lewis GK, Pazgier M, DeVico AL (2017). Survivors remorse: antibody-mediated protection against HIV-1. Immunol Rev.

[13] Radebe M, Gounder K, Mokgoro M, Ndhlovu ZM, Mncube Z, Mkhize L (2015). Broad and persistent Gag-specific CD8 + T-cell responses are associated with viral control but rarely drive viral escape during primary HIV-1 infection. AIDS.

[14] Sun J, Zhao Y, Peng Y, Han Z, Liu G, Qin L (2016). Multiple T-cell responses are associated with better control of acute HIV-1 infection. Medicine (Baltimore).

[15] Mothe B, Llano A, Ibarrondo J, Zamarreño J, Schiaulini M, Miranda C (2012). CTL responses of high functional avidity and broad variant cross-reactivity are associated with HIV control. PLoS ONE.

[16] Deng K, Pertea M, Rongvaux A, Wang L, Durand CM, Ghiaur G (2015). Broad CTL response is required to clear latent HIV-1 due to dominance of escape mutations. Nature.

[17] O’Connell KA, Brennan TP, Bailey JR, Ray SC, Siliciano RF, Blankson JN (2010). Control of HIV-1 in elite suppressors despite ongoing replication and evolution in plasma virus. J Virol.

[18] Carlson JM, Le AQ, Shahid A, Brumme ZL (2015). HIV-1 adaptation to HLA: a window into virus–host immune interactions. Trends Microbiol.

[19] Henn MR, Boutwell CL, Charlebois P, Lennon NJ, Power KA, Macalalad AR (2012). Whole genome deep sequencing of HIV-1 reveals the impact of early minor variants upon immune recognition during acute infection. PLoS Pathog.

[20] Fischer W, Ganusov VV, Giorgi EE, Hraber PT, Keele BF, Leitner T (2010). Transmission of single HIV-1 genomes and dynamics of early immune escape revealed by ultra-deep sequencing. PLoS ONE.

[21] Gounder K, Padayachi N, Mann JK, Radebe M, Mokgoro M, van der Stok M (2015). High frequency of transmitted HIV-1 Gag HLA class I-driven immune escape variants but minimal immune selection over the first year of clade C infection. PLoS ONE.

[22] Brumme ZL, Brumme CJ, Carlson J, Streeck H, John M, Eichbaum Q (2008). Marked epitope- and allele-specific differences in rates of mutation in human immunodeficiency type 1 (HIV-1) Gag, Pol, and Nef cytotoxic T-lymphocyte epitopes in acute/early HIV-1 infection. J Virol.

[23] Dilernia DA, Jones L, Rodriguez S, Turk G, Rubio AE, Pampuro S (2008). HLA-driven convergence of HIV-1 viral subtypes B and F toward the adaptation to immune responses in human populations. PLoS ONE.

[24] Juarez-Molina CI, Payne R, Soto-Nava M, Avila-Rios S, Valenzuela-Ponce H, Adland E (2014). Impact of HLA selection pressure on HIV fitness at a population level in Mexico and Barbados. J Virol.

[25] Carlson JM, Brumme ZL (2008). HIV evolution in response to HLA-restricted CTL selection pressures: a population-based perspective. Microbes Infect.

[26] Roberts HE, Hurst J, Robinson N, Brown H, Flanagan P, Vass L (2015). Structured observations reveal slow HIV-1 CTL escape. PLoS Genet.

[27] Carlson JM, Brumme CJ, Martin E, Listgarten J, Brockman MA, Le AQ (2012). Correlates of protective cellular immunity revealed by analysis of population-level immune escape pathways in HIV-1. J Virol.

[28] Bello G, Casado C, Sandonis V, Alonso-Nieto M, Vicario JL, García S (2005). A subset of human immunodeficiency virus type 1 long-term non-progressors is characterized by the unique presence of ancestral sequences in the viral population. J Gen Virol.

[29] Bello G, Casado C, Sandonis V, Alvaro-Cifuentes T, Dos Santos CAR, García S (2007). Plasma viral load threshold for sustaining intrahost HIV type 1 evolution. AIDS Res Hum Retroviruses.

[30] Immonen TT, Leitner T (2014). Reduced evolutionary rates in HIV-1 reveal extensive latency periods among replicating lineages. Retrovirology.

[31] Bailey JR, Brennan TP, O’Connell KA, Siliciano RF, Blankson JN (2009). Evidence of CD8 + T-cell-mediated selective pressure on human immunodeficiency virus type 1 nef in HLA-B*57 + elite suppressors. J Virol.

[32] Salgado M, Brennan TP, O’Connell KA, Bailey JR, Ray SC, Siliciano RF (2010). Evolution of the HIV-1 nef gene in HLA-B*57 positive elite suppressors. Retrovirology.

[33] Saez-Cirion A, Lacabaratz C, Lambotte O, Versmisse P, Urrutia A, Boufassa F (2007). HIV controllers exhibit potent CD8 T cell capacity to suppress HIV infection ex vivo and peculiar cytotoxic T lymphocyte activation phenotype. Proc Natl Acad Sci.

[34] Saez-Cirion A, Sinet M, Shin SY, Urrutia A, Versmisse P, Lacabaratz C (2009). Heterogeneity in HIV suppression by CD8 T cells from HIV controllers: association with Gag-specific CD8 T cell responses. J Immunol.

[35] O’Connell KA, Hegarty RW, Siliciano RF, Blankson JN (2011). Viral suppression of multiple escape mutants by de novo CD8(+) T cell responses in a human immunodeficiency virus-1 infected elite suppressor. Retrovirology.

[36] Pohlmeyer CW, Buckheit RW, Siliciano RF, Blankson JN (2013). CD8 + T cells from HLA-B*57 elite suppressors effectively suppress replication of HIV-1 escape mutants. Retrovirology.

[37] Miura T, Brockman MA, Schneidewind A, Lobritz M, Pereyra F, Rathod A (2009). HLA-B57/B*5801 human immunodeficiency virus type 1 elite controllers select for rare gag variants associated with reduced viral replication capacity and strong cytotoxic T-lymphotye recognition. J Virol.

[38] Bailey JR, Zhang H, Wegweiser BW, Yang H, Herrera L, Ahonkhai A (2007). Evolution of HIV-1 in an HLA-B*57-positive patient during virologic escape. J Infect Dis.

[39] Goulder PJ, Phillips RE, Colbert RA, McAdam S, Ogg G, Nowak MA (1997). Late escape from an immunodominant cytotoxic T-lymphocyte response associated with progression to AIDS. Nat Med.

[40] Feeney ME, Tang Y, Roosevelt KA, Leslie AJ, McIntosh K, Karthas N (2004). Immune escape precedes breakthrough human immunodeficiency virus type 1 viremia and broadening of the cytotoxic T-lymphocyte response in an HLA-B27-positive long-term-nonprogressing child. J Virol.

[41] Metzner KJ, Scherrer AU, Von Wyl V, Böni J, Yerly S, Klimkait T (2014). Limited clinical benefit of minority K103 N and Y181C-variant detection in addition to routine genotypic resistance testing in antiretroviral therapy-naive patients. AIDS.

[42] Cozzi-Lepri A, Noguera-Julian M, Di Giallonardo F, Schuurman R, Däumer M, Aitken S (2015). Low-frequency drug-resistant HIV-1 and risk of virological failure to first-line NNRTI-based ART: a multicohort European case-control study using centralized ultrasensitive 454 pyrosequencing. J Antimicrob Chemother.

[43] Casadellà M, Manzardo C, Noguera-Julian M, Ferrer E, Domingo P, Pérez-Álvarez S (2015). Clinical value of ultradeep HIV-1 genotyping and tropism testing in late presenters with advanced disease. AIDS.

[44] Swenson LC, Däumer M, Paredes R (2012). Next-generation sequencing to assess HIV tropism. Curr Opin HIV AIDS.

[45] Côrtes FH, Bello G, Vorsatz C, Pilotto JH, Guimarães ML, Grinsztejn B (2013). Higher cross-subtype IFN-γ ELISpot responses to Gag and Nef peptides in Brazilian HIV-1 subtype B- and F1- than in C-infected subjects. Vaccine.

[46] Sharkey M, Babic DZ, Greenough T, Gulick R, Kuritzkes DR, Stevenson M (2011). Episomal viral cDNAS identify a reservoir that fuels viral rebound after treatment interruption and that contributes to treatment failure. PLoS Pathog.

[47] Babraham Bioinformatics. FastQC—a quality control tool for high throughput sequence data. 2015.

[48] Bolger AM, Lohse M, Usadel B (2014). Trimmomatic: a flexible trimmer for Illumina sequence data. Bioinformatics.

[49] Joshi N, Fass J. Sickle: A sliding-window, adaptive, quality-based trimming tool for FastQ files. 2011.

[50] Kearse M, Moir R, Wilson A, Stones-Havas S, Cheung M, Sturrock S (2012). Geneious basic: an integrated and extendable desktop software platform for the organization and analysis of sequence data. Bioinformatics.

[51] Li H, Handsaker B, Wysoker A, Fennell T, Ruan J, Homer N (2009). The sequence alignment/map format and SAMtools. Bioinformatics.

[52] Darriba D, Taboada GL, Doallo R, Posada D (2012). jModelTest 2: more models, new heuristics and parallel computing. Nat Methods.

[53] Los Alamos National Laboratory. Los Alamos Immunology Database. 2015. http://www.hiv.lanl.gov/content/immunology. Accessed 29 Sept 2017.

[54] Torres AJL, Angelo ALD, Silva MO, de Bastos MC, de Souza DF, Inocêncio LA (2013). Establishing the reference range for T lymphocytes subpopulations in adults and children from Brazil. Rev Inst Med Trop Sao Paulo.

[55] Teixeira SLM, de Sá NBR, Campos DP, Coelho AB, Guimarães ML, Leite TCNF (2014). Association of the HLA-B*52 allele with non-progression to AIDS in Brazilian HIV-1-infected individuals. Genes Immun.

[56] Assone T, Paiva A, Fonseca LAM, Casseb J (2016). Genetic markers of the host in persons living with HTLV-1, HIV and HCV infections. Viruses.

[57] Zaunders J, van Bockel D (2013). Innate and adaptive immunity in long-term non-progression in HIV disease. Front Immunol.

[58] Côrtes FH, Passaes CPB, Bello G, Teixeira SLM, Vorsatz C, Babic D (2015). HIV controllers with different viral load cutoff levels have distinct virologic and immunologic profiles. JAIDS J Acquir Immune Defic Syndr.

[59] Mens H, Kearney M, Wiegand A, Shao W, Schønning K, Gerstoft J (2010). HIV-1 continues to replicate and evolve in patients with natural control of HIV infection. J Virol.

[60] Okulicz JF (2012). Elite controllers and long-term nonprogressors: models for HIV vaccine development?. J AIDS Clin Res.

[61] Gaardbo JC, Hartling HJ, Ronit A, Thorsteinsson K, Madsen HO, Springborg K (2013). Different immunological phenotypes associated with preserved CD4 + T cell counts in HIV-infected controllers and viremic long term non-progressors. PLoS ONE.

[62] Perreau M, Levy Y, Pantaleo G (2013). Immune response to HIV. Curr Opin HIV AIDS.

[63] Gijsbers EF, Feenstra KA, van Nuenen AC, Navis M, Heringa J, Schuitemaker H (2013). HIV-1 replication fitness of HLA-B*57/58:01 CTL escape variants is restored by the accumulation of compensatory mutations in gag. PLoS ONE.

[64] Altfeld M, Addo MM, Rosenberg ES, Hecht FM, Lee PK, Vogel M (2003). Influence of HLA-B57 on clinical presentation and viral control during acute HIV-1 infection. AIDS.

[65] Bailey JR, Williams TM, Siliciano RF, Blankson JN (2006). Maintenance of viral suppression in HIV-1-infected HLA-B*57 + elite suppressors despite CTL escape mutations. J Exp Med.

[66] Boutwell CL, Rowley CF, Essex M (2009). Reduced viral replication capacity of human immunodeficiency virus type 1 subtype C caused by cytotoxic-T-lymphocyte escape mutations in HLA-B57 epitopes of capsid protein. J Virol.

[67] Hatano H, Delwart EL, Norris PJ, Lee T-H, Dunn-Williams J, Hunt PW (2009). Evidence for persistent low-level viremia in individuals who control human immunodeficiency virus in the absence of antiretroviral therapy. J Virol.

[68] Pereyra F, Palmer S, Miura T, Block BL, Wiegand A, Rothchild AC (2009). Persistent low-level viremia in HIV-1 elite controllers and relationship to immunologic parameters. J Infect Dis.

[69] de Azevedo SSD, Caetano DG, Côrtes FH, Teixeira SLM, dos Santos Silva K, Hoagland B (2017). Highly divergent patterns of genetic diversity and evolution in proviral quasispecies from HIV controllers. Retrovirology.

[70] Gijsbers EF, Kootstra NA, Setiawan LC, van Nuenen AC (2015). Viral evolution in HLA-B27-restricted CTL epitopes in human immunodeficiency virus type 1-infected individuals. J Gen Virol.

[71] Starcich BR, Hahn BH, Shaw GM, McNeely PD, Modrow S, Wolf H (1986). Identification and characterization of conserved and variable regions in the envelope gene of HTLV-III/LAV, the retrovirus of AIDS. Cell.

[72] Yebra G, Brown AJL. Evolutionary rates of HIV 1 accessory genes from full length datasets across subtypes. 22nd HIV Dyn Evol Work. 2015.

[73] De Azevedo SSD, Caetano DG, Côrtes FH, Teixeira SLM, Dos K, Silva S (2017). Highly divergent patterns of genetic diversity and evolution in proviral quasispecies from HIV controllers. Retrovirology.

[74] Hughes AL, Becker EA, Lauck M, Karl JA, Braasch AT, O’Connor DH (2012). SIV genome-wide pyrosequencing provides a comprehensive and unbiased view of variation within and outside CD8 T lymphocyte epitopes. PLoS ONE.

[75] Mudd PA, Ericsen AJ, Burwitz BJ, Wilson NA, O’Connor DH, Hughes AL (2012). Escape from CD8(+) T cell responses in Mamu-B*00801(+) macaques differentiates progressors from elite controllers. J Immunol.

[76] Cale EM, Hraber P, Giorgi EE, Fischer W, Bhattacharya T, Leitner T (2011). Epitope-specific CD8 + T lymphocytes cross-recognize mutant simian immunodeficiency virus (SIV) sequences but fail to contain very early evolution and eventual fixation of epitope escape mutations during SIV infection. J Virol.

[77] O’Connell KA, Brennan TP, Bailey JR, Ray SC, Siliciano RF, Blankson JN (2010). Control of HIV-1 in elite suppressors despite ongoing replication and evolution in plasma virus. J Virol.

[78] Migueles SA, Laborico AC, Imamichi H, Shupert WL, Royce C, McLaughlin M (2003). The differential ability of HLA B*5701 + long-term nonprogressors and progressors to restrict human immunodeficiency virus replication is not caused by loss of recognition of autologous viral gag sequences. J Virol.

[79] Bailey JR, Brennan TP, O’Connell KA, Siliciano RF, Blankson JN (2009). Evidence of CD8 + T-cell-mediated selective pressure on human immunodeficiency virus type 1 nef in HLA-B*57 + elite suppressors. J Virol.

[80] Cale EM, Hraber P, Giorgi EE, Fischer W, Bhattacharya T, Leitner T (2011). Epitope-specific CD8 + T lymphocytes cross-recognize mutant simian immunodeficiency virus (SIV) sequences but fail to contain very early evolution and eventual fixation of epitope escape mutations during SIV infection. J Virol.

[81] Goulder PJR, Watkins DI (2004). HIV and SIV CTL escape: implications for vaccine design. Nat Rev Immunol.

[82] Fryer HR, Frater J, Duda A, Palmer D, Phillips RE, McLean AR (2012). Cytotoxic T-lymphocyte escape mutations identified by HLA association favor those which escape and revert rapidly. J Virol.

[83] Sunshine JE, Larsen BB, Maust B, Casey E, Deng W, Chen L (2015). Fitness-balanced escape determines resolution of dynamic founder virus escape processes in HIV-1 infection. J Virol.

[84] Martin E, Carlson JM, Le AQ, Chopera DR, McGovern R, Rahman MA (2014). Early immune adaptation in HIV-1 revealed by population-level approaches. Retrovirology.

[85] Leslie AJ, Pfafferott KJ, Chetty P, Draenert R, Addo MM, Feeney M (2004). HIV evolution: CTL escape mutation and reversion after transmission. Nat Med.

[86] Roberts HE, Hurst J, Robinson N, Brown H, Flanagan P, Vass L (2015). Structured observations reveal slow HIV-1 CTL escape. PLoS Genet.

