# Supplementary material for: Next-generation sequencing analyses of the emergence and maintenance of mutations in CTL epitopes in HIV controllers with differential viremia control
Source: Retrovirology. 2018 Sep 10;15:62. doi: 10.1186/s12977-018-0444-z (PMC6131818; doi:10.1186/s12977-018-0444-z)
Supplement: Supplementary file 1 — Additional file 1.Table S1. Primer set used in the present study. Table S2. Gag Epitopes selected for study according to the HLA-B alleles carried by the HICs. Table S3. Nef Epitopes selected for study according to the HLA-B alleles carried by the HICs. Table S4. NGS mapping and coverage statistics of gag and nef distributed according to the patients. Table S5. Full list of Gag mutations and associated epitopes recognized by HLA alleles carried by the HICs. Table S6. Full list of Nef mutations and associated epitopes recognized by HLA alleles carried by the HICs [file 12977_2018_444_MOESM1_ESM.docx]

**Table S1. Primer sets used on the present study**

| **Gene** | **Position** | **Direction** | **Name** | **Sequence** |
| --- | --- | --- | --- | --- |
| *gag* | Outer primers | Forward | SCAOSD | GGGACTTTCCGCTGGGGACTTTC |
|  |  | Reverse | G17 | TCCACATTTCCAACAGCCYTTTTT |
|  | Inner Primers | Forward | SCANSD | CGAGCCCTCAGATGCTGCATATAAGC |
|  |  | Reverse | P24-1 | CCCTGRCATGCTGTCATCA |
| *nef* | Outer primers | Forward | NEF-1 | CCTGTGCCTCTTCAGCTACCACCG |
|  |  | Reverse | SCDOAD | AGTCACACAACAGACGGGCACACAC |
|  | Inner Primers | Forward | NEF-3 | TGRACAGATAGRRTYATAGAA |
|  |  | Reverse | SCDNAD | ACGTGCCCTCAAGGCAAGCTTTATTGAGGCT |

**Table S2. Gag Epitopes^a^ selected for study according to the HLA-B alleles carried by HICs**

| **Epitope** | **Position ^b^** | **Sequence** | **HLA associated** |
| --- | --- | --- | --- |
| GI9 | 11 - 19 | GELDRWEKI | B40 |
| KK9 | 114 - 122 | KTQQAAADK | B57 |
| QV9 | 127 - 135 | QVSQNYPIV | B14 |
|  |  |  | B15 |
| LI10 | 138 - 147 | LQGQMVHQAI | B51 |
| GI8 | 140 - 147 | GQMVHQAI | B48 |
|  |  |  | B51 |
| HL9 | 144 - 152 | HQAISPRTL | B15 |
|  |  |  | B42 |
| QW11 | 145 - 155 | QAISPRTLNAW | B57 |
| IW9 | 147 - 155 | ISPRTLNAW | B15 |
|  |  |  | B58 |
| SV9 | 148 - 156 | SPRTLNAWV | B07 |
|  |  |  | B81 |
| VF9 | 156 - 164 | VKVVEEKAF | B15 |
| II11 | 159 - 169 | IEEKAFSPEVI | B45 |
| EV9 | 160 - 168 | EEKAFSPEV | B14 |
|  |  |  | B42 |
|  |  |  | B44 |
|  |  |  | B81 |
| KF11 | 162 - 172 | KAFSPEVIPMF | B48 |
|  |  |  | B51 |
|  |  |  | B57 |
|  |  |  | B58 |
| VL8 | 168 - 175 | VIPMFSAL | B15 |
| LL10 | 175 - 184 | LSEGATPQDL | B44 |
| SL9 | 176 - 184 | SEGATPQDL | B40 |
| AL10 | 179 - 188 | ATPQDLNTML | B58 |
| TM8 | 180 - 187 | TPQDLNTM | B81 |
| TL9 | 180 - 188 | TPQDLNTML | B07 |
|  |  |  | B14 |
|  |  |  | B40 |
|  |  |  | B42 |
| KK10 | 18 - 27 | KIRLRPGGKK | B07 |
| DV9 | 183 - 191 | DLNMMLNIV | B14 |
| IK9 | 19 - 27 | IRLRPGGKK | B81 |
| GL9 | 193 - 201 | GHQAAMQML |  |
|  |  |  | B15 |
|  |  |  | B42 |
|  |  |  | B52 |
| KA9 | 202 - 210 | KETINEEAA | B40 |
| RY10 | 20 - 29 | RLRPGGKKKY | B15 |
| RY10 | 20 - 29 | RLRPGGKKKY | B44 |
| EL9 | 207 - 215 | EEAAEWDRL | B40 |
| AV9 | 210 - 218 | AEWDRLHPV | B40 |
| RM9 | 22 - 30 | RPGGKKHYM | B07 |
|  |  |  | B42 |
| RL10 | 22 - 31 | RPGGKKKYKL | B51 |
| GI11 | 226 - 236 | GQMREPRGSDI | B15 |
| TW10 | 240 - 249 | TSTLQEQIGW | B57 |
|  |  |  | B58 |
| KK10 | 263 - 272 | KRWIILGLNK | B81 |
| GY9 | 269 - 277 | GLNKIVRMY | B15 |
| VI9 | 274 - 282 | VRMYSPVSI | B07 |
|  |  |  | B15 |
|  |  |  | B57 |
| RI8 | 275 - 282 | RMYSPTSI | B52 |
| FF9 | 293 - 301 | FRDYVDRFF | B42 |
| RL11 | 294 - 304 | RDYVDRFYKTL | B44 |
| YL9 | 296 - 304 | YVDRFFKTL | B15 |
| DA9 | 298 - 306 | DRFFKTLRA | B14 |
| RV9 | 305 - 313 | RAEQASQEV | B14 |
|  |  |  | B40 |
|  |  |  | B45 |
|  |  |  | B51 |
|  |  |  | B49 |
| AW11 | 306 - 316 | AEQASQDVKNW | B15 |
|  |  |  | B44 |
| QW9 | 308 - 316 | QATQDVKNW | B57 |
| Q 10 | 308 - 316 | QASQEVKNW | B58 |
| VL9 | 313 - 321 | VKNWMTETL | B48 |
|  |  |  | B51 |
| NI9 | 325 - 333 | NANPDCKTI | B51 |
| DL9 | 329 - 337 | DCKTILKAL | B14 |
|  |  |  | B15 |
| LF11 | 34 - 44 | LVWASRELERF | B52 |
|  |  |  | B57 |
| GK8 | 352 - 359 | GVGGPGHK | B51 |
| GL9 | 355 - 363 | GPGHKARVL | B07 |
| AS10 | 364 - 373 | AEAMSQVTNS | B40 |
|  |  |  | B42 |
|  |  |  | B45 |
|  |  |  | B81 |
| WF9 | 36 - 44 | WASRELERF | B42 |
|  |  |  | B51 |
| CC9 | 405 - 413 | CRAPRKKGC | B14 |
| TL8 | 427 - 434 | TERQANFL | B40 |
| RI9 | 429 - 437 | RQANFLGKI | B15 |
|  |  |  | B48 |
|  |  |  | B51 |
|  |  |  | B52 |
| KL9 | 481 - 489 | KELYPLTSL | B40 |
| GY9 | 71 - 79 | GSEELRSLY | B07 |
| EV10 | 73 - 82 | EELRSLYNTV | B40 |
| EV9 | 74 - 82 | ELRSLYNTV | B40 |
|  |  |  | B52 |
| RY11 | 76 - 86 | RSLYNTVATLY | B57 |
|  |  |  | B58 |
| LY9 | 78 - 86 | LYNTVATLY | B44 |
| IL10 | 92 - 101 | IEIKDTKEAL | B40 |
|  |  |  | B42 |

^a^ as available at Los Alamos Immunology database; ^b^ Related to the HXB2 translated amino acid sequence

**Table S3. Nef Epitopes^a^ selected for study according to the HLA-B alleles carried by HICs**

| **Epitope** | **Position ^b^** | **Sequence** | **HLA associated** |
| --- | --- | --- | --- |
| QW9 | 105 - 113 | QRQDILDLW | B14 |
| RY11 | 105 - 115 | RRQDILDLWIY | B07 |
| KY11 | 105 - 115 | KRQEILDLWVY | B44 |
|  |  |  | B57 |
|  |  |  | B58 |
| RI9 | 106 - 114 | RQDILDLWI | B15 |
| HW9 | 116 - 124 | HTQGYFPDW | B15 |
|  |  |  | B57 |
|  |  |  | B58 |
| TY11 | 117 - 127 | TQGYFPDWQNY | B15 |
|  |  |  | B81 |
| YT9 | 120 - 128 | YFPDWQNYT | B51 |
|  |  |  | B57 |
|  |  |  | B49 |
| YY9 | 127 - 135 | YTPGPGIRY | B58 |
|  |  |  | B57 |
| TL10 | 128 - 137 | TPGPGVRYPL | B07 |
|  |  |  | B42 |
| WM8 | 13 - 20 | WPTVRERM | B15 |
| YF9 | 135 - 143 | YPLTFGWCF | B58 |
| PL10 | 136 - 145 | PLTFGWCYKL | B07 |
|  |  |  | B49 |
| LL9 | 137 - 145 | LTFGWCFKL | B15 |
|  |  |  | B57 |
| VL10 | 180 - 189 | VLEWRFDSRL | B40 |
| WF9 | 183 - 191 | WRFDSRLAF | B14 |
|  |  |  | B15 |
| WH10 | 183 - 192 | WRFDSRLAFH | B81 |
| DV9 | 186 - 194 | DSRLAFHHV | B51 |
| RR9 | 188 - 196 | RLAFHHVAR | B52 |
| AK9 | 190 - 198 | AFHHVAREK | B51 |
| RA9 | 19 - 27 | RMRRAEPAA | B07 |
|  |  |  | B15 |
|  |  |  | B58 |
| LS9 | 37 - 45 | LEKHGAITS | B40 |
| EV11 | 64 - 74 | EEVGFPVKPQV | B45 |
| FL9 | 68 - 76 | FPVTPQVPL | B07 |
| RM9 | 71 - 79 | TPQVPLRPM | B07 |
|  |  |  | B42 |
|  |  |  | B81 |
| PY10 | 72 - 81 | PQVPLRPMTY | B51 |
| VY8 | 74 - 81 | VPLRPMTY | B07 |
| PK8 | 75 - 82 | PLRPMTYK | B15 |
| RL9 | 77 - 85 | RPMTYKGAL | B07 |
| RV9 | 77 - 85 | RPMTYKAAV | B42 |
| KF9 | 82 - 90 | KAAFDLSFF | B58 |
|  |  |  | B57 |
| AL9 | 83 - 91 | AAVDLSHFL | B07 |
|  |  |  | B42 |
|  |  |  | B81 |
| AK9 | 84 - 92 | AVDLSHFLK | B15 |
| FL8 | 90 - 97 | FLKEKGGL | B07 |
|  |  |  | B40 |
|  |  |  | B57 |
| KL9 | 92 - 100 | KEKGGLEGL | B40 |
|  |  |  | B44 |

^a^ as available at Los Alamos Immunology database; ^b^ Related to the HXB2 translated amino acid sequence

**Table S4. NGS mapping and coverage statistics of *gag* and *nef* distributed according to patients**

| **Patient** | **Visit** | **Number of Reads** |  | ***gag*** | |  | ***nef*** | |
| --- | --- | --- | --- | --- | --- | --- | --- | --- |
|  |  |  |  | **Median Coverage/bp (IQR)** | **Consensus Size (bp)** |  | **Median Coverage/bp (IQR)** | **Consensus Size (bp)** |
| EC02 | V_E_ | 5.003.182 |  | 313.475 | 1238 |  | 481.172 | 821 |
|  |  |  |  | (185.803 – 496.466) |  |  | (426.211 – 563.297) |  |
|  | V_L_ | 3.505.033 |  | 278.739 | 1115 |  | 261.540 | 821 |
|  |  |  |  | (224.901 – 378.457) |  |  | (187.512 – 347.389) |  |
| EC52 | V_E_ | 3.711.980 |  | 117.678 | 1233 |  | 411.984 | 821 |
|  |  |  |  | (77.763 – 174.696) |  |  | (335.414 – 503.150) |  |
|  | V_L_ | 3.228.117 |  | 265.931 | 1084 |  | 269.424 | 821 |
|  |  |  |  | (207.848 – 344.114) |  |  | (220.768 – 333.011) |  |
| EC17 | V_E_ | NA |  | NA | NA |  | NA | NA |
|  |  |  |  |  |  |  |  |  |
|  | V_L_ | 3.521.212 |  | 334.115 | 1113 |  | 100.458 | 821 |
|  |  |  |  | (193.692 – 491.751) |  |  | (63.477 – 135.286) |  |
| EC11 | V_E_ | 3.968.360 |  | 259.633 | 1095 |  | 478.446 | 821 |
|  |  |  |  | (142.248 – 378.219) |  |  | (434.887 – 534.803) |  |
|  | V_L_ | 2.385.946 |  | 195.481 | 1062 |  | 283.701 | 821 |
|  |  |  |  | (135.711 – 273.048) |  |  | (264.096 – 312.581) |  |
| EC42 | V_E_ | 1.684.093 |  | NA | NA |  | 64.325 | 821 |
|  |  |  |  |  |  |  | (56.038 – 71.145) |  |
|  | V_L_ | 2.482.542 |  | 204.823 | 1054 |  | 229.115 | 821 |
|  |  |  |  | (150.037 – 276.734) |  |  | (207.804 – 258.725) |  |
| EC18 | V_E_ | 4.325.987 |  | 308.385 | 1188 |  | 299.952 | 821 |
|  |  |  |  | (105.213 – 535.980) |  |  | (276.520 – 329.667) |  |
|  | V_L_ | 3.238.646 |  | 183.912 | 1213 |  | 426.834 | 821 |
|  |  |  |  | (63.292 – 272.838) |  |  | (400.251 – 478.804) |  |
| VC06 | V_E_ | 3.756.561 |  | 365.832 | 1098 |  | NA | NA |
|  |  |  |  | (139.187 – 571.490) |  |  |  |  |
|  | V_L_ | 3.010.452 |  | 249.588 | 1162 |  | 123.688 | 821 |
|  |  |  |  | (138.606 – 399.083) |  |  | (109.534 – 140.590) |  |
| VC10 | V_E_ | 4.226.988 |  | 140.795 | 1244 |  | 586.848 | 821 |
|  |  |  |  | (57.552 – 225.829) |  |  | (543.124 – 646.658) |  |
|  | V_L_ | 2.783.816 |  | 228.698 | 1241 |  | 207.286 | 821 |
|  |  |  |  | (68.823 – 364.659) |  |  | (192.997 – 233.045) |  |
| VC14 | V_E_ | 861.491 |  | 70.910 | 1069 |  | 65.595 | 821 |
|  |  |  |  | (51.944 – 90.448) |  |  | (63.448 – 78.992) |  |
|  | V_L_ | 2.391.996 |  | 246.708 | 1117 |  | 18.498 | 821 |
|  |  |  |  | (205.839 – 332.977) |  |  | (17.311 – 21.300) |  |
| VC15 | V_E_ | 3.389.165 |  | 184.682 | 1235 |  | 456.541 | 821 |
|  |  |  |  | (86.656 – 297.503) |  |  | (421.875 – 517.799) |  |
|  | V_L_ | 2.321.730 |  | 122.896 | 1246 |  | 323.997 | 821 |
|  |  |  |  | (82.646 – 173.119) |  |  | (281.137 – 386.994) |  |
| VC16 | V_E_ | 3.758.835 |  | 111.700 | 1232 |  | 611.240 | 821 |
|  |  |  |  | (62.243 – 172.716) |  |  | (473.587 – 753.073) |  |
|  | V_L_ | 3.014.330 |  | 214.445 | 1252 |  | 236.389 | 821 |
|  |  |  |  | (69.671 – 329.870) |  |  | (133.982 – 332.199) |  |

NA – Not Available

**Table S5. Full list of Gag mutations and associated epitopes recognized by HLA alleles carried by HICs**

| **Patient** | **Mutation** |  | **Frequency** | |  | **Epitope** | | | |
| --- | --- | --- | --- | --- | --- | --- | --- | --- | --- |
|  |  |  | **V_E_** | **V_L_** |  | **HLA** | **Position** | **Sequence** | **Location** |
| EC02 | W36L |  | 0,6% | 0,0% |  | B52 | 34-44 | LVWASRELERF | I |
|  | R39M |  | 0,6% | 0,0% |  | B52 | 34-44 | LVWASRELERF | I |
|  | L41P |  | 0,0% | 0,5% |  | B52 | 34-44 | LVWASRELERF | I |
|  | V46A |  | 0,0% | 0,5% |  | B52 | 34-44 | LVWASRELERF | A |
|  | S72P |  | 0,0% | 0,8% |  | B52 | 74-82 | ELRSLYNTV | A |
|  | A83S |  | 0,0% | 22,9% |  | B52 | 74-82 | ELRSLYNTV | A |
|  | L85I |  | 0,7% | 0,0% |  | B52 | 74-82 | ELRSLYNTV | A |
|  | N137S |  | 0,0% | 3,0% |  | B48 | 140-147 | GQMVHQAI | A |
|  | I147V |  | 0,0% | 0,6% |  | B48 | 140-147 | GQMVHQAI | I |
|  | G192W |  | 0,6% | 0,0% |  | B52 | 193-201 | GHQAAMQML | A |
|  | G192V |  | 0,6% | 0,0% |  | B52 | 193-201 | GHQAAMQML | A |
|  | R275G |  | 0,0% | 0,8% |  | B52 | 275-282 | RMYSPTSI | I |
|  | S278C |  | 0,0% | 22,8% |  | B52 | 275-282 | RMYSPTSI | I |
|  | T280A |  | 0,6% | 0,0% |  | B52 | 275-282 | RMYSPTSI | I |
| VC06 | Q136R |  | 0,0% | 1,3% |  | B15 | 127-135 | QVSQNYPIV | A |
|  | L138P |  | 0,6% | 0,0% |  | B48 | 140-147 | GQMVHQAI | A |
|  | Q139R |  | 0,8% | 0,6% |  | B48 | 140-147 | GQMVHQAI | A |
|  | E160G |  | 0,6% | 0,0% |  | B15 / B48 | 156-164 / 162-172 | VKVVEEKAF / KAFSPEVIPMF | I / A |
|  | I169V |  | 0,0% | 0,5% |  | B48 / B15 | 162-172 / 168-175 | KAFSPEVIPMF / VIPMFSAL | I |
|  | I169M |  | 1,4% | 0,0% |  | B15 | 162-172 / 168-175 | KAFSPEVIPMF / VIPMFSAL | I |
|  | D235G |  | 0,0% | 0,5% |  | B15 | 226-236 | GQMREPRGSDI | I |
|  | I236V |  | 0,7% | 0,0% |  | B15 | 226-236 | GQMREPRGSDI | I |
|  | D295G |  | 0,0% | 1,6% |  | B15 | 296-304 | YVDRFFKTL | A |
|  | F300L |  | 3,2% | 0,0% |  | B15 | 296-304 | YVDRFFKTL | I |
|  | R302I |  | 0,0% | 0,8% |  | B15 | 296-304 | YVDRFFKTL | I |
|  | E307G |  | 1,4% | 0,0% |  | B15 | 306-316 | AEQASQDVKNW | I |
|  | Q308H |  | 0,0% | 1,2% |  | B15 | 306-316 | AEQASQDVKNW | I |
|  | Q311R |  | 0,0% | 0,5% |  | B15 / B48 | 306-316 / 313-321 | AEQASQDVKNW / VKNWMTETL | I / A |
|  | E312G |  | 0,0% | 0,5% |  | B15 / B48 | 306-316 / 313-321 | AEQASQDVKNW / VKNWMTETL | I / A |
|  | T318A |  | 0,0% | 0,6% |  | B15 / B48 | 306-316 / 313-321 | AEQASQDVKNW / VKNWMTETL | I |
|  | E319G |  | 0,0% | 2,5% |  | B15 / B48 | 306-316 / 313-321 | AEQASQDVKNW / VKNWMTETL | I |
|  | K331R |  | 0,6% | 0,0% |  | B15 | 329-337 | DCKTILKAL | I |
|  | T332A |  | 0,0% | 0,5% |  | B15 | 329-337 | DCKTILKAL | I |
| EC52 | R43L |  | 0,0% | 0,8% |  | B57 | 34-44 | LVWASRELERF | I |
|  | F44L |  | 0,0% | 0,7% |  | B57 | 34-44 | LVWASRELERF | I |
|  | R150I |  | 0,0% | 0,6% |  | B57 | 145-155 | QAISPRTLNAW | I |
|  | S165G |  | 0,0% | 0,7% |  | B45 / B57 | 159-169 / 162-172 | IEEKAFSPEVI / KAFSPEVIPMF | I |
|  | I169V |  | 0,8% | 0,0% |  | B45 / B57 | 159-169 / 162-172 | IEEKAFSPEVI / KAFSPEVIPMF | I |
|  | G238V |  | 0,0% | 0,6% |  | B57 | 240-249 | TSTLQEQIGW | A |
|  | G248E |  | 1,0% | 0,0% |  | B57 | 240-249 | TSTLQEQIGW | I |
|  | M250I |  | 0,0% | 0,9% |  | B57 | 240-249 | TSTLQEQIGW | A |
|  | I273V |  | 0,0% | 0,7% |  | B57 | 274-282 | VRMYSPVSI | A |
|  | R275K |  | 0,8% | 0,0% |  | B57 | 274-282 | VRMYSPVSI | I |
|  | S281G |  | 0,0% | 0,6% |  | B57 | 274-282 | VRMYSPVSI | I |
|  | I285V |  | 0,0% | 0,7% |  | B57 | 274-282 | VRMYSPVSI | A |
|  | L304I |  | 0,0% | 0,5% |  | B45 | 305-313 | RAEQASQEV | A |
|  | E312G |  | 0,7% | 0,0% |  | B45 / B57 | 305-313 / 308-316 | RAEQASQEV / QATQDVKNW | I |
|  | M317I |  | 0,0% | 0,7% |  | B57 | 308-316 | QATQDVKNW | A |
| VC10 | R20S |  | 0,5% | 0,0% |  | B15 | 20-29 | RLRPGGKKKY | I |
|  | K26S |  | 0,6% | 0,0% |  | B15 | 20-29 | RLRPGGKKKY | I |
|  | S72P |  | 0,6% | 0,0% |  | B52 | 74-82 | ELRSLYNTV | A |
|  | R76K |  | 0,0% | 0,9% |  | B52 | 74-82 | ELRSLYNTV | I |
|  | V82L |  | 0,6% | 0,0% |  | B52 | 74-82 | ELRSLYNTV | I |
|  | A83V |  | 0,0% | 0,9% |  | B52 | 74-82 | ELRSLYNTV | A |
|  | N126S |  | 0,7% | 94,2% |  | B15 | 127-135 | QVSQNYPIV | A |
|  | N126S |  | 0,0% | 5,6% |  | B15 | 127-135 | QVSQNYPIV | A |
|  | N137S |  | 0,0% | 0,7% |  | B15 | 127-135 | QVSQNYPIV | A |
|  | M142V |  | 3,1% | 0,0% |  | B15 | 144-152 | HQAISPRTL | A |
|  | V143A |  | 0,6% | 0,0% |  | B15 | 144-152 | HQAISPRTL | A |
|  | A146P |  | 39,9% | 37,6% |  | B15 | 144-152 / 147-155 | HQAISPRTL / ISPRTLNAW | I / A |
|  | A146S |  | 59,3% | 62,4% |  | B15 | 144-152 / 147-155 | HQAISPRTL / ISPRTLNAW | I / A |
|  | W155R |  | 0,0% | 0,7% |  | B15 | 147-155 / 156-164 | ISPRTLNAW / VKVVEEKAF | I / A |
|  | V159I |  | 0,6% | 1,4% |  | B15 | 156-164 | VKVVEEKAF | I |
|  | F172L |  | 0,0% | 0,7% |  | B15 | 168-175 | VIPMFSAL | I |
|  | S173T |  | 43,1% | 67,5% |  | B15 | 168-175 | VIPMFSAL | I |
|  | G192E |  | 0,0% | 1,1% |  | B15 / B52 | 193-201 | GHQAAMQML | A |
|  | E203D |  | 0,0% | 1,5% |  | B15 / B52 | 193-201 | GHQAAMQML | A |
|  | I223V |  | 41,6% | 32,4% |  | B15 | 226-236 | GQMREPRGSDI | A |
|  | R232K |  | 0,0% | 0,9% |  | B15 | 226-236 | GQMREPRGSDI | I |
|  | G233R |  | 0,0% | 0,9% |  | B15 | 226-236 | GQMREPRGSDI | I |
|  | N271S |  | 0,7% | 0,0% |  | B15 | 269-277 | GLNKIVRMY | I |
|  | M276I |  | 0,0% | 1,3% |  | B15 / B15 / B52 | 269-277 / 274-282 / 275-282 | GLNKIVRMY / VRMYSPTSI / RMYSPTSI | I |
|  | S278G |  | 0,0% | 0,6% |  | B15 / B15 / B52 | 269-277 / 274-282 / 275-282 | GLNKIVRMY / VRMYSPTSI / RMYSPTSI | A / I |
|  | T280V |  | 0,7% | 2,1% |  | B15 / B15 / B52 | 269-277 / 274-282 / 275-282 | GLNKIVRMY / VRMYSPTSI / RMYSPTSI | A / I |
|  | T280S |  | 45,3% | 66,7% |  | B15 / B52 | 274-282 / 275-282 | VRMYSPTSI / RMYSPTSI | A / I |
|  | T280I |  | 52,7% | 17,4% |  | B15 / B52 | 274-282 / 275-282 | VRMYSPTSI / RMYSPTSI | A / I |
|  | S281G |  | 0,0% | 2,4% |  | B15 / B52 | 274-282 / 275-282 | VRMYSPTSI / RMYSPTSI | I |
|  | F300L |  | 0,5% | 0,0% |  | B15 | 296-304 | YVDRFFKTL | I |
|  | E307G |  | 0,0% | 0,5% |  | B15 | 296-304 / 306-316 | YVDRFFKTL / AEQASQDVKNW | A / I |
|  | S310T |  | 0,7% | 0,0% |  | B15 | 306-316 | AEQASQDVKNW | I |
|  | Q311R |  | 0,0% | 0,6% |  | B15 | 306-316 | AEQASQDVKNW | I |
|  | N327D |  | 0,0% | 0,7% |  | B15 | 329-337 | DCKTILKAL | A |
|  | D329G |  | 0,0% | 0,6% |  | B15 | 329-337 | DCKTILKAL | I |
|  | K331R |  | 0,7% | 0,0% |  | B15 | 329-337 | DCKTILKAL | I |
| EC11 | K26R |  | 0,0% | 94,1% |  | B81 | 19-27 | IRLRPGGKK | I |
|  | T186A |  | 0,5% | 0,0% |  | B81 | 180-187 | TPQDLNTM | I |
|  | R264G |  | 0,6% | 0,0% |  | B81 | 263-272 | KRWIILGLNK | I |
|  | I267V |  | 0,8% | 0,0% |  | B81 | 263-272 | KRWIILGLNK | I |
|  | Q308R |  | 2,3% | 0,0% |  | B49 | 305-313 | RAEQASQEV | I |
|  | E312G |  | 0,8% | 2,1% |  | B49 | 305-313 | RAEQASQEV | I |
| EC42 | R39K |  | R* | 6,2% |  | B51 | 36-44 | WASRELERF | I |
|  | E40Q |  | E* | 17,7% |  | B51 | 36-44 | WASRELERF | I |
|  | N126S |  | S* | 79,9% |  | B15 | 127-135 | QVSQNYPIV | A |
|  | N126R |  | S* | 21,0% |  | B15 | 127-135 | QVSQNYPIV | A |
|  | M142I |  | M* | 9,4% |  | B51 | 140-147 | GQMVHQAI | I |
|  | I169V |  | I* | 0,6% |  | B51 | 162-172 | KAFSPEVIPMF | I |
|  | S176P |  | S* | 2,4% |  | B15 | 168-175 | VIPMFSAL | A |
|  | G178R |  | G* | 9,7% |  | B15 | 168-175 | VIPMFSAL | A |
|  | G192R |  | G* | 9,8% |  | B15 | 193-201 | GHQAAMQML | A |
|  | S234G |  | S* | 0,5% |  | B15 | 226-236 | GQMREPRGSDI | I |
|  | D284G |  | D* | 0,7% |  | B15 | 274-282 | VRMYSPTSI | A |
|  | Q311R |  | Q* | 1,0% |  | B51 | 305-313 | RAEQASQEV | I |
|  | T318A |  | T* | 0,8% |  | B15 | 306-316 | AEQASQDVKNW | A |
|  | T320A |  | T* | 0,5% |  | B51 | 313-321 | VKNWMTETL | I |
|  | N325D |  | N* | 1,0% |  | B51 | 325-333 | NANPDCKTI | I |
|  | G338R |  | G* | 7,2% |  | B15 | 329-337 | DCKTILKAL | A |
| VC14 | R20L |  | 1,0% | 0,0% |  | B44 / B42 | 20-29 / 22-30 | RLRPGGKKKY / RPGGKKHYM | A / I |
|  | G25V |  | 0,6% | 0,0% |  | B44 / B42 | 20-29 / 22-30 | RLRPGGKKKY / RPGGKKHYM | I |
|  | K26N |  | 0,7% | 0,0% |  | B44 / B42 | 20-29 / 22-30 | RLRPGGKKKY / RPGGKKHYM | I |
|  | K30I |  | 0,9% | 0,0% |  | B44 / B42 | 20-29 / 22-30 | RLRPGGKKKY / RPGGKKHYM | A / I |
|  | L31I |  | 1,3% | 0,0% |  | B44 / B42 | 20-29 / 22-30 | RLRPGGKKKY / RPGGKKHYM | A / I |
|  | W36L |  | 0,9% | 0,0% |  | B42 | 36-44 | WASRELERF | I |
|  | R39M |  | 0,6% | 0,0% |  | B42 | 36-44 | WASRELERF | I |
|  | R39K |  | 0,0% | 1,2% |  | B42 | 36-44 | WASRELERF | I |
|  | R43L |  | 2,1% | 0,0% |  | B42 | 36-44 | WASRELERF | I |
|  | N47Y |  | 2,4% | 0,0% |  | B42 | 36-44 | WASRELERF | A |
|  | R76I |  | 0,5% | 0,0% |  | B44 | 78-86 | LYNTVATLY | A |
|  | V82I |  | 99,7% | 77,9% |  | B44 | 78-86 | LYNTVATLY | I |
|  | C87Y |  | 97,4% | 99,7% |  | B44 | 78-86 | LYNTVATLY | A |
|  | H89R |  | 0,0% | 0,6% |  | B42 | 92-101 | IEIKDTKEAL | A |
|  | Q90L |  | 0,8% | 0,0% |  | B42 | 92-101 | IEIKDTKEAL | A |
|  | R91G |  | 0,0% | 0,5% |  | B42 | 92-101 | IEIKDTKEAL | A |
|  | R91M |  | 0,7% | 0,0% |  | B42 | 92-101 | IEIKDTKEAL | A |
|  | R91S |  | 0,6% | 0,0% |  | B42 | 92-101 | IEIKDTKEAL | A |
|  | E93D |  | 0,6% | 0,0% |  | B42 | 92-101 | IEIKDTKEAL | I |
|  | D96Y |  | 0,9% | 0,0% |  | B42 | 92-101 | IEIKDTKEAL | I |
|  | D96G |  | 0,6% | 0,0% |  | B42 | 92-101 | IEIKDTKEAL | I |
|  | K98N |  | 0,6% | 0,0% |  | B42 | 92-101 | IEIKDTKEAL | I |
|  | M142I |  | 1,1% | 0,0% |  | B42 | 144-152 | HQAISPRTL | A |
|  | Q145K |  | 0,7% | 0,0% |  | B42 | 144-152 | HQAISPRTL | I |
|  | I147L |  | 99,7% | 81,1% |  | B42 | 144-152 | HQAISPRTL | I |
|  | S148P |  | 0,0% | 0,5% |  | B42 | 144-152 | HQAISPRTL | I |
|  | R150I |  | 1,3% | 0,0% |  | B42 | 144-152 | HQAISPRTL | I |
|  | W155L |  | 0,7% | 0,0% |  | B42 | 144-152 | HQAISPRTL | A |
|  | P166Q |  | 0,6% | 0,0% |  | B42/B44 | 160-168 | EEKAFSPEV | I |
|  | P181T |  | 1,1% | 0,0% |  | B44 | 175-184 | LSEGATPQDL | I |
|  | P181Q |  | 0,6% | 0,0% |  | B42 | 180-188 | TPQDLNTML | I |
|  | D183Y |  | 0,8% | 0,0% |  | B42 | 180-188 | TPQDLNTML | I |
|  | G193V |  | 1,1% | 0,0% |  | B42 | 193-201 | GHQAAMQML | I |
|  | Q195K |  | 0,8% | 0,0% |  | B42 | 193-201 | GHQAAMQML | I |
|  | E203Y |  | 0,7% | 0,0% |  | B42 | 193-201 | GHQAAMQML | A |
|  | E203G |  | 0,0% | 1,0% |  | B42 | 193-201 | GHQAAMQML | A |
|  | R294I |  | 0,5% | 0,0% |  | B42 / B44 | 293-301 / 294-304 | FRDYVDRFF / RDYVDRFYKTL | I |
|  | D295Y |  | 1,4% | 0,0% |  | B42 / B44 | 293-301 / 294-304 | FRDYVDRFF / RDYVDRFYKTL | I |
|  | D295G |  | 0,0% | 1,7% |  | B42 / B44 | 293-301 / 294-304 | FRDYVDRFF / RDYVDRFYKTL | I |
|  | D298Y |  | 1,1% | 0,0% |  | B42 / B44 | 293-301 / 294-304 | FRDYVDRFF / RDYVDRFYKTL | I |
|  | D298G |  | 1,2% | 0,0% |  | B42 / B44 | 293-301 / 294-304 | FRDYVDRFF / RDYVDRFYKTL | I |
|  | F300L |  | 0,8% | 0,0% |  | B42 / B44 | 293-301 / 294-304 | FRDYVDRFF / RDYVDRFYKTL | I |
|  | R305I |  | 0,8% | 0,0% |  | B44 / B44 | 294-304 / 306-316 | RDYVDRFYKTL / AEQASQDVKNW | A |
|  | S310T |  | 99,7% | 81,1% |  | B44 | 306-316 | AEQASQDVKNW | I |
|  | E312G |  | 0,0% | 0,6% |  | B44 | 306-316 | AEQASQDVKNW | I |
|  | W316L |  | 0,8% | 0,0% |  | B44 | 306-316 | AEQASQDVKNW | I |
|  | M317I |  | 1,8% | 0,0% |  | B44 | 306-316 | AEQASQDVKNW | A |
|  | E319Y |  | 0,7% | 0,0% |  | B44 | 306-316 | AEQASQDVKNW | A |
| VC15 | I34M |  | 0,5% | 0,0% |  | B57 | 34-44 | LVWASRELERF | I |
|  | S38G |  | 3,1% | 0,0% |  | B57 | 34-44 | LVWASRELERF | I |
|  | V82I |  | 43,4% | 84,9% |  | B57 | 76-86 | RSLYNTVATLY | I |
|  | S111G |  | 0,0% | 1,0% |  | B57 | 114-122 | KTQQAAADK | A |
|  | S111N |  | 0,0% | 3,1% |  | B57 | 114-122 | KTQQAAADK | A |
|  | K113E |  | 1,4% | 0,0% |  | B57 | 114-122 | KTQQAAADK | A |
|  | A115T |  | 0,0% | 4,4% |  | B57 | 114-122 | KTQQAAADK | I |
|  | A115V |  | 11,1% | 2,5% |  | B57 | 114-122 | KTQQAAADK | I |
|  | A119T |  | 10,3% | 0,0% |  | B57 | 114-122 | KTQQAAADK | I |
|  | A120Q |  | 1,0% | 0,0% |  | B57 | 114-122 | KTQQAAADK | I |
|  | D121A |  | 30,6% | 81,8% |  | B57 | 114-122 | KTQQAAADK | I |
|  | T122A |  | 49,2% | 82,1% |  | B57 | 114-122 | KTQQAAADK | I |
|  | H124N |  | 59,7% | 0,0% |  | B57 | 114-122 | KTQQAAADK | A |
|  | H124S |  | 0,8% | 0,0% |  | B57 | 114-122 | KTQQAAADK | A |
|  | H124N |  | 0,0% | 8,2% |  | B57 | 114-122 | KTQQAAADK | A |
|  | H124Y |  | 0,0% | 0,6% |  | B57 | 114-122 | KTQQAAADK | A |
|  | H124R |  | 2,4% | 3,7% |  | B57 | 114-122 | KTQQAAADK | A |
|  | Q145R |  | 0,6% | 0,0% |  | B57 | 145-155 | QAISPRTLNAW | I |
|  | I147Y |  | 0,0% | 3,6% |  | B57 | 145-155 | QAISPRTLNAW | I |
|  | S148K |  | 0,0% | 3,5% |  | B57 | 145-155 | QAISPRTLNAW | I |
|  | P149T |  | 0,0% | 0,6% |  | B57 | 145-155 | QAISPRTLNAW | I |
|  | P149R |  | 0,0% | 3,1% |  | B57 | 145-155 | QAISPRTLNAW | I |
|  | T151A |  | 0,8% | 0,0% |  | B57 | 145-155 | QAISPRTLNAW | I |
|  | T151K |  | 0,0% | 3,0% |  | B57 | 145-155 | QAISPRTLNAW | I |
|  | N153S |  | 0,0% | 0,6% |  | B57 | 145-155 | QAISPRTLNAW | I |
|  | A154G |  | 0,0% | 2,5% |  | B57 | 145-155 | QAISPRTLNAW | I |
|  | V156Q |  | 0,0% | 2,4% |  | B57 | 145-155 | QAISPRTLNAW | A |
|  | V158P |  | 0,0% | 2,1% |  | B57 | 145-155 | QAISPRTLNAW | A |
|  | V159C |  | 0,0% | 2,0% |  | B57 | 162-172 | KAFSPEVIPMF | A |
|  | E160W |  | 0,0% | 1,9% |  | B57 | 162-172 | KAFSPEVIPMF | A |
|  | E161S |  | 0,0% | 1,9% |  | B57 | 162-172 | KAFSPEVIPMF | A |
|  | A163S |  | 0,0% | 7,4% |  | B57 | 162-172 | KAFSPEVIPMF | I |
|  | N271T |  | 0,0% | 24,7% |  | B57 | 274-282 | VRMYSPVSI | A |
|  | N271K |  | 0,0% | 0,9% |  | B57 | 274-282 | VRMYSPVSI | A |
|  | T280V |  | 1,8% | 98,8% |  | B57 | 274-282 | VRMYSPVSI | I |
|  | D284G |  | 0,5% | 0,0% |  | B57 | 274-282 | VRMYSPVSI | A |
|  | S310P |  | 0,0% | 0,5% |  | B57 | 308-316 | QATQDVKNW | I |
| VC16 | I34L |  | 32,2% | 0,0% |  | B57 | 34-44 | LVWASRELERF | I |
|  | V35I |  | 32,6% | 0,0% |  | B57 | 34-44 | LVWASRELERF | I |
|  | W36R |  | 0,0% | 0,5% |  | B57 | 34-44 | LVWASRELERF | I |
|  | R39K |  | 0,0% | 5,6% |  | B57 | 34-44 | LVWASRELERF | I |
|  | V46I |  | 29,3% | 0,0% |  | B57 | 34-44 | LVWASRELERF | A |
|  | V82L |  | 25,9% | 0,0% |  | B57 | 76-86 | RSLYNTVATLY | I |
|  | S111G |  | 0,0% | 1,4% |  | B57 | 114-122 | KTQQAAADK | A |
|  | A115E |  | 25,0% | 0,0% |  | B57 | 114-122 | KTQQAAADK | I |
|  | A118P |  | 24,9% | 0,0% |  | B57 | 114-122 | KTQQAAADK | I |
|  | A119T |  | 24,9% | 0,0% |  | B57 | 114-122 | KTQQAAADK | I |
|  | T122A |  | 4,8% | 71,6% |  | B57 | 114-122 | KTQQAAADK | I |
|  | G123K |  | 24,7% | 0,0% |  | B57 | 114-122 | KTQQAAADK | A |
|  | G123A |  | 0,0% | 2,2% |  | B57 | 114-122 | KTQQAAADK | A |
|  | H124N |  | 99,1% | 64,4% |  | B14 | 127-135 | QVSQNYPIV | A |
|  | H124S |  | 0,7% | 35,3% |  | B14 | 127-135 | QVSQNYPIV | A |
|  | S125R |  | 30,9% | 0,0% |  | B14 | 127-135 | QVSQNYPIV | A |
|  | N126R |  | 10,2% | 0,0% |  | B14 | 127-135 | QVSQNYPIV | A |
|  | Q127R |  | 0,0% | 1,2% |  | B14 | 127-135 | QVSQNYPIV | I |
|  | Q127H |  | 76,7% | 98,3% |  | B14 | 127-135 | QVSQNYPIV | I |
|  | Y132C |  | 0,6% | 0,0% |  | B14 | 127-135 | QVSQNYPIV | I |
|  | I138L |  | 75,5% | 94,1% |  | B14 | 127-135 | QVSQNYPIV | A |
|  | I138L |  | 0,0% | 5,3% |  | B14 | 127-135 | QVSQNYPIV | A |
|  | A146P |  | 74,6% | 99,4% |  | B57 | 145-155 | QAISPRTLNAW | I |
|  | T151A |  | 0,0% | 0,6% |  | B57 | 145-155 | QAISPRTLNAW | I |
|  | V159I |  | 22,0% | 9,3% |  | B14 / B57 | 160-168 / 162-172 | EEKAFSPEV / KAFSPEVIPMF | A |
|  | E160K |  | 0,0% | 19,3% |  | B14 / B57 | 160-168 / 162-172 | EEKAFSPEV / KAFSPEVIPMF | I / A |
|  | A163G |  | 78,3% | 75,4% |  | B14 / B57 | 160-168 / 162-172 | EEKAFSPEV / KAFSPEVIPMF | I |
|  | S165D |  | 0,6% | 0,0% |  | B14 / B57 | 160-168 / 162-172 | EEKAFSPEV / KAFSPEVIPMF | I |
|  | S165N |  | 77,6% | 75,0% |  | B14 / B57 | 160-168 / 162-172 | EEKAFSPEV / KAFSPEVIPMF | I |
|  | E167G |  | 1,5% | 0,0% |  | B14 / B57 | 160-168 / 162-172 | EEKAFSPEV / KAFSPEVIPMF | I |
|  | V168T |  | 0,0% | 22,8% |  | B14 / B57 | 160-168 / 162-172 | EEKAFSPEV / KAFSPEVIPMF | I |
|  | S173T |  | 37,7% | 74,1% |  | B57 | 162-172 | KAFSPEVIPMF | A |
|  | G178R |  | 0,0% | 5,3% |  | B14 | 180-188 | TPQDLNTML | A |
|  | T190A |  | 0,5% | 0,0% |  | B14 | 180-188 / 183-191 | TPQDLNTML / DLNMMLNIV | A /I |
|  | V191M |  | 0,5% | 0,0% |  | B14 | 183-191 | DLNMMLNIV | I |
|  | V191I |  | 33,3% | 99,4% |  | B14 | 183-191 | DLNMMLNIV | I |
|  | T242N |  | 78,4% | 99,5% |  | B57 | 240-249 | TSTLQEQIGW | I |
|  | I247V |  | 1,7% | 0,0% |  | B57 | 240-249 | TSTLQEQIGW | I |
|  | N252S |  | 32,3% | 0,0% |  | B57 | 240-249 | TSTLQEQIGW | A |
|  | K272R |  | 0,0% | 0,5% |  | B57 | 274-282 | VRMYSPVSI | A |
|  | R275K |  | 0,0% | 7,3% |  | B57 | 274-282 | VRMYSPVSI | I |
|  | T280V |  | 21,8% | 0,0% |  | B57 | 274-282 | VRMYSPVSI | I |
|  | I282T |  | 0,6% | 0,0% |  | B57 | 274-282 | VRMYSPVSI | I |
|  | I285V |  | 0,5% | 2,9% |  | B57 | 274-282 | VRMYSPVSI | A |
|  | D295N |  | 0,0% | 9,9% |  | B14 | 298-306 | DRFFKTLRA | A |
|  | D295G |  | 1,5% | 0,0% |  | B14 | 298-306 | DRFFKTLRA | A |
|  | T303A |  | 0,0% | 0,9% |  | B14 | 298-306 | DRFFKTLRA | I |
|  | E307K |  | 0,0% | 10,8% |  | B14 / B57 | 305-313 / 308-316 | RAEQASQEV / QATQDVKNW | I |
|  | S310T |  | 17,1% | 0,0% |  | B14 / B57 | 305-313 / 308-316 | RAEQASQEV / QATQDVKNW | I |
|  | E312K |  | 0,0% | 12,1% |  | B14 / B57 | 305-313 / 308-316 | RAEQASQEV / QATQDVKNW | I |
|  | V313I |  | 0,8% | 0,0% |  | B14 / B57 | 305-313 / 308-316 | RAEQASQEV / QATQDVKNW | I |
|  | N315S |  | 0,7% | 0,0% |  | B14 / B57 | 305-313 / 308-316 | RAEQASQEV / QATQDVKNW | A / I |
|  | T318A |  | 0,9% | 0,0% |  | B57 | 308-316 | QATQDVKNW | A |
|  | E319G |  | 0,9% | 0,0% |  | B57 | 308-316 | QATQDVKNW | A |
|  | A326S |  | 0,0% | 7,0% |  | B14 | 329-337 | DCKTILKAL | A |
|  | K335R |  | 49,2% | 58,9% |  | B14 | 329-337 | DCKTILKAL | I |
|  | A340G |  | 70,9% | 96,6% |  | B14 | 329-337 | DCKTILKAL | A |
| EC17 | E12K |  | 1,7% | 0,0% |  | B40 | 11-19 | GELDRWEKI | I |
|  | R15K |  | 1,8% | 0,0% |  | B40 | 11-19 | GELDRWEKI | I |
|  | W16R |  | 0,0% | 0,6% |  | B40 | 11-19 | GELDRWEKI | I |
|  | Y79F |  | 2,1% | 0,0% |  | B07 / B40 | 71-79 / 73-82 | GSEELRSLY / EELRSLYNTV | I |
|  | N80S |  | 0,0% | 3,9% |  | B07 / B40 | 71-79 / 73-82 | GSEELRSLY / EELRSLYNTV | A / I |
|  | V82A |  | 0,9% | 0,0% |  | B07 / B40 | 71-79 / 73-82 | GSEELRSLY / EELRSLYNTV | A / I |
|  | R91E |  | 1,8% | 0,0% |  | B40 | 92-101 | IEIKDTKEAL | A |
|  | E93N |  | 1,8% | 0,0% |  | B40 | 92-101 | IEIKDTKEAL | I |
|  | I94V |  | 2,0% | 0,0% |  | B40 | 92-101 | IEIKDTKEAL | I |
|  | E99K |  | 1,5% | 0,0% |  | B40 | 92-101 | IEIKDTKEAL | I |
|  | I104V |  | 2,1% | 0,0% |  | B40 | 92-101 | IEIKDTKEAL | A |
|  | T180A |  | 0,0% | 0,5% |  | B07 / B40 | 176-184 / 180-188 | SEGATPQDL / TPQDLNTML | I |
|  | D183N |  | 1,6% | 0,0% |  | B07 / B40 | 176-184 / 180-188 | SEGATPQDL / TPQDLNTML | I |
|  | N185S |  | 0,0% | 0,8% |  | B07 / B40 | 176-184 / 180-188 | SEGATPQDL / TPQDLNTML | A / I |
|  | T204A |  | 0,0% | 0,5% |  | B40 | 202-210 / 207-215 | KETINEEAA / EEAAEWDRL | I / A |
|  | E211G |  | 0,0% | 0,6% |  | B40 | 202-210 / 207-215 | KETINEEAA / EEAAEWDRL | A / I |
|  | R214K |  | 1,5% | 0,0% |  | B40 | 207-215 / 210-218 | EEAAEWDRL / AEWDRLHPV | I |
|  | N271T |  | 1,4% | 0,0% |  | B07 | 274-282 | VRMYSPVSI | A |
|  | Y277C |  | 0,0% | 0,6% |  | B07 | 274-282 | VRMYSPVSI | I |
|  | T280A |  | 0,5% | 0,0% |  | B07 | 274-282 | VRMYSPVSI | I |
|  | S281G |  | 0,0% | 1,2% |  | B07 | 274-282 | VRMYSPVSI | I |
|  | D284N |  | 1,4% | 0,0% |  | B07 | 274-282 | VRMYSPVSI | A |
|  | I285R |  | 0,0% | 0,7% |  | B07 | 274-282 | VRMYSPVSI | A |
|  | R305K |  | 1,6% | 0,0% |  | B40 | 305-313 | RAEQASQEV | I |
|  | E307G |  | 0,0% | 0,6% |  | B40 | 305-313 | RAEQASQEV | I |
| EC18 | K28Q |  | 33,9% | 0,0% |  | B07 | 22-30 | RPGGKKHYM | I |
|  | K28R |  | 65,9% | 99,6% |  | B07 | 22-30 | RPGGKKHYM | I |
|  | I34L |  | 21,0% | 99,8% |  | B52 | 34-44 | LVWASRELERF | I |
|  | S38G |  | 0,0% | 0,6% |  | B52 | 34-44 | LVWASRELERF | I |
|  | R39K |  | 0,0% | 99,5% |  | B52 | 34-44 | LVWASRELERF | I |
|  | R43Q |  | 17,2% | 0,0% |  | B52 | 34-44 | LVWASRELERF | I |
|  | R76K |  | 20,2% | 0,0% |  | B07 | 71-79 | GSEELRSLY | I |
|  | T84A |  | 3,1% | 0,0% |  | B52 | 74-82 | ELRSLYNTV | A |
|  | L85P |  | 1,0% | 0,5% |  | B52 | 74-82 | ELRSLYNTV | A |
|  | I147M |  | 82,8% | 99,8% |  | B07 | 148-156 | SPRTLNAWV | A |
|  | S148P |  | 0,0% | 0,6% |  | B07 | 148-156 | SPRTLNAWV | I |
|  | N153D |  | 0,0% | 2,0% |  | B07 | 148-156 | SPRTLNAWV | I |
|  | N189H |  | 13,6% | 0,0% |  | B07 | 180-188 | TPQDLNTML | A |
|  | N271A |  | 0,0% | 0,7% |  | B07 / B52 | 274-282 / 275-282 | VRMYSPVSI / RMYSPTSI | A |
|  | I273V |  | 0,0% | 1,1% |  | B07 / B52 | 274-282 / 275-282 | VRMYSPVSI / RMYSPTSI | A |
|  | I273M |  | 2,8% | 0,0% |  | B07 / B52 | 274-282 / 275-282 | VRMYSPVSI / RMYSPTSI | A |
|  | R275K |  | 18,7% | 0,0% |  | B07 / B52 | 274-282 / 275-282 | VRMYSPVSI / RMYSPTSI | A / I |
|  | T280A |  | 79,7% | 0,0% |  | B07 / B52 | 274-282 / 275-282 | VRMYSPVSI / RMYSPTSI | I |
|  | T280V |  | 19,9% | 0,0% |  | B07 / B52 | 274-282 / 275-282 | VRMYSPVSI / RMYSPTSI | I |
|  | T280S |  | 0,0% | 99,7% |  | B07 / B52 | 274-282 / 275-282 | VRMYSPVSI / RMYSPTSI | I |
|  | S281C |  | 16,0% | 0,0% |  | B07 / B52 | 274-282 / 275-282 | VRMYSPVSI / RMYSPTSI | I |

* Consensus amino acid from available bulk sequence available showed shown instead of frequency; Location A - Adjacent to Epitope; Location I - Within Epitope

**Table S6. Full list of Nef mutations and associated epitopes recognized by HLA alleles carried by HICs**

| **Patient** | **Mutation** |  | **Frequency** | |  | **Epitope** | | | |
| --- | --- | --- | --- | --- | --- | --- | --- | --- | --- |
|  |  |  | **V_E_** | **V_L_** |  | **HLA** | **Position** | **Sequence** | **Location** |
| EC02 | D186G |  | 0,8% | 0,0% |  | B52 | 188-196 | RLAFHHVAR | A |
|  | S187G |  | 0,0% | 0,8% |  | B52 | 188-196 | RLAFHHVAR | A |
|  | E197G |  | 0,0% | 0,5% |  | B52 | 188-196 | RLAFHHVAR | A |
| EC11 | K82R |  | 0,0% | 0,5% |  | B81 | 71-79 / 83-91 | RPQVPLRPM / AAVDLSHFL | A |
|  | I114A |  | 0,6% | 0,0% |  | B81 | 117-127 | TQGYFPDWQNY | A |
|  | D123G |  | 0,5% | 0,0% |  | B81 / B49 | 117-127 / 120-128 | TQGYFPDWQNY / YFPDWQNYT | I |
|  | V133P |  | 70,7% | 99,4% |  | B49 | 136-145 | PLTFGWCYKL | A |
|  | G140R |  | 4,2% | 0,0% |  | B49 | 136-145 | PLTFGWCYKL | I |
|  | K144M |  | 0,0% | 1,4% |  | B49 | 136-145 | PLTFGWCYKL | I |
|  | R184E |  | 1,7% | 0,0% |  | B81 | 183-192 | WRFDSRLAFH | I |
|  | D186G |  | 0,5% | 0,0% |  | B81 | 183-192 | WRFDSRLAFH | I |
|  | F191V |  | 85,5% | 60,2% |  | B81 | 183-192 | WRFDSRLAFH | I |
|  | V194I |  | 4,8% | 0,0% |  | B81 | 183-192 | WRFDSRLAFH | A |
| EC42 | V10M |  | 0,5% | 0,0% |  | B15 | 13-20 | WPAIRERM | A |
|  | V10R |  | 99,0% | 8,4% |  | B15 | 13-20 | WPAIRERM | A |
|  | V10K |  | 0,0% | 91,0% |  | B15 | 13-20 | WPAIRERM | A |
|  | I11V |  | 0,5% | 0,0% |  | B15 | 13-20 | WPAIRERM | A |
|  | G12R |  | 0,0% | 90,4% |  | B15 | 13-20 | WPAIRERM | A |
|  | M79I |  | 0,0% | 89,6% |  | B15 / B51 | 75-82 / 72-81 | PLRPMTYK / PQVPLRPMTY | I |
|  | K82R |  | 0,7% | 0,0% |  | B15 / B51 | 75-82 / 72-81 | PLRPMTYK / PQVPLRPMTY | I/ A |
|  | A83G |  | 0,5% | 10,2% |  | B15 / B51 / B15 | 75-82 / 72-81 / 84-92 | PLRPMTYK / PQVPLRPMTY / AVDLSHFLK | A |
|  | V85L |  | 0,0% | 10,7% |  | B15 | 84-92 | AVDLSHFLK | I |
|  | H89R |  | 0,0% | 0,5% |  | B15 | 84-92 | AVDLSHFLK | I |
|  | F90L |  | 0,0% | 0,5% |  | B15 | 84-92 | AVDLSHFLK | I |
|  | R106G |  | 0,0% | 0,7% |  | B15 | 106-114 | RQDILDLWI | I |
|  | D108G |  | 0,0% | 0,7% |  | B15 | 106-114 | RQDILDLWI | I |
|  | Y115H |  | 1,7% | 0,0% |  | B15 | 106-114 / 116-124 | RQDILDLWI / HTQGYFPDW | A |
|  | H116N |  | 0,6% | 0,0% |  | B15 | 106-114 / 116-124 / 117-127 | RQDILDLWI / HTQGYFPDW / TQGYFPDWQNY | A / I |
|  | H116R |  | 0,0% | 0,6% |  | B15 | 106-114 / 116-124 / 117-127 | RQDILDLWI / HTQGYFPDW / TQGYFPDWQNY | A / I |
|  | T117A |  | 0,0% | 0,5% |  | B15 / B15 / B51 | 116-124 / 117-127 / 120-128 | HTQGYFPDW / TQGYFPDWQNY / YFPDWQNYT | I / A |
|  | N126C |  | 0,0% | 9,9% |  | B15 / B15 / B51 | 116-124 / 117-127 / 120-128 | HTQGYFPDW / TQGYFPDWQNY / YFPDWQNYT | I |
|  | N126S |  | 99,2% | 0,0% |  | B15 / B15 / B51 | 116-124 / 117-127 / 120-128 | HTQGYFPDW / TQGYFPDWQNY / YFPDWQNYT | A / I |
|  | Y135F |  | 99,2% | 10,4% |  | B15 | 137-145 | LTFGWCFKL | A |
|  | T138A |  | 0,0% | 0,6% |  | B15 | 137-145 | LTFGWCFKL | I |
|  | G140R |  | 0,0% | 88,5% |  | B15 | 137-145 | LTFGWCFKL | I |
|  | Y143L |  | 0,0% | 0,6% |  | B15 | 137-145 | LTFGWCFKL | I |
|  | V148I |  | 99,2% | 0,7% |  | B15 | 137-145 | LTFGWCFKL | A |
|  | F185L |  | 0,7% | 0,0% |  | B15 / B51 | 183-191 / 186-194 | WRFDSRLAF / DSRLAFHHV | I / A |
|  | D186G |  | 0,0% | 0,5% |  | B15 / B51 | 183-191 / 186-194 | WRFDSRLAF / DSRLAFHHV | I |
|  | R188S |  | 25,1% | 0,0% |  | B15 / B51 / B51 | 183-191 / 186-194 / 190-198 | WRFDSRLAF / DSRLAFHHV / AFHHVAREK | I / A |
|  | F191S |  | 0,0% | 0,7% |  | B15 / B51 / B51 | 183-191 / 186-194 / 190-198 | WRFDSRLAF / DSRLAFHHV / AFHHVAREK | I |
|  | E197G |  | 0,0% | 0,5% |  | B51 | 186-194 / 190-198 | DSRLAFHHV / AFHHVAREK | A / I |
|  | L198R |  | 0,0% | 0,5% |  | B51 | 190-198 | AFHHVAREK | I |
|  | L198E |  | 0,0% | 0,5% |  | B51 | 190-198 | AFHHVAREK | I |
| EC18 | D28G |  | 0,0% | 2,5% |  | B07 | 19-27 | RMRRAEPAA | A |
|  | M79V |  | 0,0% | 0,6% |  | B07 | 68-76 / 71-79 / 74-81 / 77-85 | FPVTPQVPL / TPQVPLRPM / VPLRPMTY / RPMTYKGAL | A / I |
|  | M79I |  | 0,0% | 0,6% |  | B07 | 68-76 / 71-79 / 74-81 / 77-85 | FPVTPQVPL / TPQVPLRPM / VPLRPMTY / RPMTYKGAL | A / I |
|  | L87P |  | 1,2% | 0,0% |  | B07 | 83-91 / 90-97 | RPMTYKGAL / AAVDLSHFL / FLKEKGGL | A / I |
|  | K92R |  | 0,0% | 68,5% |  | B07 | 83-91 / 90-97 | AAVDLSHFL / FLKEKGGL | A / I |
|  | G96R |  | 0,0% | 6,9% |  | B07 | 90-97 | FLKEKGGL | I |
|  | S103P |  | 0,5% | 0,0% |  | B07 | 105-115 | RRQDILDLWIY | A |
|  | D108G |  | 0,5% | 0,0% |  | B07 | 105-115 | RRQDILDLWIY | I |
|  | V133A |  | 0,6% | 0,0% |  | B07 | 128-137 / 136-145 | TPGPGVRYPL / PLTFGWCYKL | I / A |
|  | R134G |  | 0,0% | 2,6% |  | B07 | 128-137 / 136-145 | TPGPGVRYPL / PLTFGWCYKL | I / A |
|  | T138A |  | 0,0% | 0,8% |  | B07 | 128-137 / 136-145 | TPGPGVRYPL / PLTFGWCYKL | A / I |
|  | F185L |  | 0,0% | 0,8% |  | B52 | 188-196 | RLAFHHVAR | A |
|  | S187G |  | 0,7% | 0,0% |  | B52 | 188-196 | RLAFHHVAR | A |
|  | L198M |  | 0,6% | 0,0% |  | B52 | 188-196 | RLAFHHVAR | A |
|  | L198E |  | 0,0% | 0,5% |  | B52 | 188-196 | RLAFHHVAR | A |
| EC52 | T71I |  | 0,0% | 0,6% |  | B45 | 64-74 | EEVGFPVKPQV | I |
|  | M79I |  | 0,0% | 1,2% |  | B57 | 82-90 | KAAFDLSFF | A |
|  | T80A |  | 0,0% | 0,7% |  | B57 | 82-90 | KAAFDLSFF | A |
|  | V85L |  | 0,0% | 1,6% |  | B57 | 82-90 | KAAFDLSFF | I |
|  | H89R |  | 0,7% | 0,0% |  | B57 | 82-90 / 90-97 | KAAFDLSFF / FLKEKGGL | I / A |
|  | G96V |  | 0,0% | 0,8% |  | B57 | 90-97 | FLKEKGGL | I |
|  | S103Y |  | 0,0% | 0,5% |  | B57 | 105-115 | KRQEILDLWVY | A |
|  | D111Y |  | 0,0% | 0,6% |  | B57 | 105-115 | KRQEILDLWVY | I |
|  | T117A |  | 0,0% | 0,9% |  | B57 | 116-124 / 120-128 | HTQGYFPDW / YFPDWQNYT | I / A |
|  | F121L |  | 0,6% | 0,0% |  | B57 | 116-124 / 120-128 | HTQGYFPDW / YFPDWQNYT | I |
|  | Q125H |  | 0,0% | 0,6% |  | B57 | 116-124 / 120-128 / 127-135 | HTQGYFPDW / YFPDWQNYT / YTPGPGIRY | A / I |
|  | Y127C |  | 0,9% | 0,0% |  | B57 | 116-124 / 120-128 / 127-135 | HTQGYFPDW / YFPDWQNYT / YTPGPGIRY | A / I |
|  | Y143L |  | 0,0% | 0,6% |  | B57 | 137-145 | LTFGWCFKL | I |
|  | P147Q |  | 0,0% | 0,5% |  | B57 | 137-145 | LTFGWCFKL | A |
| VC06 | V74I |  | V | 13,8% |  | B15 | 75-82 | PLRPMTYK | A |
|  | A84S |  | A | 14,3% |  | B15 | 75-82 / 84-92 | PLRPMTYK / AVDLSHFLK | A / I |
|  | V85I |  | V | 45,2% |  | B15 | 75-82 / 84-92 | PLRPMTYK / AVDLSHFLK | A / I |
|  | V85L |  | V | 28,3% |  | B15 | 75-82 / 84-92 | PLRPMTYK / AVDLSHFLK | A / I |
|  | L87I |  | M | 56,9% |  | B15 | 84-92 | AVDLSHFLK | I |
|  | L87M |  | M | 13,5% |  | B15 | 84-92 | AVDLSHFLK | I |
|  | S103P |  | S | 0,9% |  | B15 | 106-114 | RQDILDLWI | A |
|  | R105K |  | R | 74,7% |  | B15 | 106-114 | RQDILDLWI | A |
|  | R106G |  | R | 0,8% |  | B15 | 106-114 | RQDILDLWI | I |
|  | I114V |  | I | 60,3% |  | B15 | 116-124 / 117-127 | HTQGYFPDW / TQGYFPDWQNY | A |
|  | T117A |  | T | 0,6% |  | B15 | 116-124 / 117-127 | HTQGYFPDW / TQGYFPDWQNY | I |
|  | T128A |  | T | 0,6% |  | B15 | 117-127 | TQGYFPDWQNY | A |
|  | L137P |  | L | 0,6% |  | B15 | 137-145 | LTFGWCFKL | I |
|  | L145P |  | L | 0,6% |  | B15 | 137-145 | LTFGWCFKL | I |
|  | D186G |  | D | 0,6% |  | B15 | 183-191 | WRFDSRLAF | I |
|  | F191S |  | F | 0,6% |  | B15 | 183-191 | WRFDSRLAF | I |
|  | V194M |  | M | 85,8% |  | B15 | 183-191 | WRFDSRLAF | A |
| VC10 | I11S |  | 0,0% | 0,8% |  | B15 | 13-20 | WPTVRERM | A |
|  | G12R |  | 0,0% | 0,6% |  | B15 | 13-20 | WPTVRERM | A |
|  | T15A |  | 59,2% | 98,5% |  | B15 | 13-20 | WPTVRERM | I |
|  | T15A |  | 0,0% | 1,3% |  | B15 | 13-20 | WPTVRERM | I |
|  | R17K |  | 0,0% | 0,6% |  | B15 | 13-20 / 19-27 | WPTVRERM / RMRRAEPAA | I / A |
|  | M20I |  | 0,0% | 0,7% |  | B15 | 13-20 / 19-27 | WPTVRERM / RMRRAEPAA | I |
|  | R21G |  | 0,0% | 0,6% |  | B15 | 13-20 / 19-27 | WPTVRERM / RMRRAEPAA | A / I |
|  | R22Q |  | 0,0% | 3,7% |  | B15 | 13-20 / 19-27 | WPTVRERM / RMRRAEPAA | A / I |
|  | E24K |  | 0,0% | 0,7% |  | B15 | 19-27 | RMRRAEPAA | I |
|  | D28N |  | 0,0% | 0,6% |  | B15 | 19-27 | RMRRAEPAA | A |
|  | R77K |  | 0,0% | 1,4% |  | B15 | 75-82 | PLRPMTYK | I |
|  | M79T |  | 0,0% | 3,0% |  | B15 | 75-82 | PLRPMTYK | I |
|  | M79I |  | 0,0% | 1,4% |  | B15 | 75-82 | PLRPMTYK | I |
|  | D86N |  | 0,0% | 1,6% |  | B15 | 84-92 | AVDLSHFLK | I |
|  | S88R |  | 4,5% | 0,0% |  | B15 | 84-92 | AVDLSHFLK | I |
|  | E93G |  | 0,0% | 1,2% |  | B15 | 84-92 | AVDLSHFLK | A |
|  | R106G |  | 0,8% | 0,0% |  | B15 | 106-114 | RQDILDLWI | I |
|  | R106G |  | 0,0% | 0,7% |  | B15 | 106-114 | RQDILDLWI | I |
|  | Q107R |  | 0,0% | 7,1% |  | B15 | 106-114 | RQDILDLWI | I |
|  | D108N |  | 0,0% | 0,7% |  | B15 | 106-114 | RQDILDLWI | I |
|  | D111N |  | 0,0% | 1,4% |  | B15 | 106-114 | RQDILDLWI | I |
|  | D123N |  | 0,0% | 0,9% |  | B15 | 116-124 | HTQGYFPDW | I |
|  | Q125R |  | 0,0% | 1,2% |  | B15 | 117-127 | TQGYFPDWQNY | I |
|  | R134K |  | 0,0% | 0,9% |  | B15 | 137-145 | LTFGWCFKL | A |
|  | Y135F |  | 0,0% | 7,2% |  | B15 | 137-145 | LTFGWCFKL | A |
|  | G140E |  | 0,0% | 1,0% |  | B15 | 137-145 | LTFGWCFKL | I |
|  | Y143L |  | 0,0% | 0,5% |  | B15 | 137-145 | LTFGWCFKL | I |
|  | E182Q |  | 99,9% | 17,8% |  | B15 | 183-191 | WRFDSRLAF | A |
|  | E182L |  | 0,0% | 81,8% |  | B15 | 183-191 | WRFDSRLAF | A |
|  | R184K |  | 0,0% | 6,3% |  | B15 | 183-191 | WRFDSRLAF | I |
|  | D186N |  | 0,0% | 1,0% |  | B52 | 183-191 / 188-196 | WRFDSRLAF / RLAFHHVAR | I / A |
|  | S187R |  | 0,0% | 0,6% |  | B52 | 183-191 / 188-196 | WRFDSRLAF / RLAFHHVAR | I / A |
|  | R188G |  | 99,8% | 90,9% |  | B52 | 183-191 / 188-196 | WRFDSRLAF / RLAFHHVAR | I |
|  | L189P |  | 0,0% | 0,5% |  | B52 | 183-191 / 188-196 | WRFDSRLAF / RLAFHHVAR | I |
|  | H192Q |  | 0,0% | 1,5% |  | B52 | 183-191 / 188-196 | WRFDSRLAF / RLAFHHVAR | A / I |
|  | E197L |  | 1,5% | 0,0% |  | B52 | 188-196 | RLAFHHVAR | A |
|  | E197K |  | 0,0% | 0,5% |  | B52 | 188-196 | RLAFHHVAR | A |
|  | E197V |  | 0,5% | 0,0% |  | B52 | 188-196 | RLAFHHVAR | A |
|  | E197G |  | 0,0% | 0,5% |  | B52 | 188-196 | RLAFHHVAR | A |
|  | H199T |  | 2,1% | 0,0% |  | B52 | 188-196 | RLAFHHVAR | A |
| VC14 | F68L |  | 0,5% | 0,5% |  | B42 | 71-79 | RPQVPLRPM | A |
|  | P69T |  | 0,6% | 0,0% |  | B42 | 71-79 | RPQVPLRPM | A |
|  | V70A |  | 0,0% | 0,6% |  | B42 | 71-79 | RPQVPLRPM | A |
|  | T71R |  | 94,5% | 3,3% |  | B42 | 71-79 | RPQVPLRPM | I |
|  | T71I |  | 0,9% | 0,0% |  | B42 | 71-79 | RPQVPLRPM | I |
|  | P78Q |  | 0,7% | 0,0% |  | B42 | 71-79 / 77-85 | RPQVPLRPM / RPMTYKAAV | I |
|  | M79I |  | 2,7% | 0,0% |  | B42 | 71-79 / 77-85 | RPQVPLRPM / RPMTYKAAV | I |
|  | K82N |  | 0,5% | 0,0% |  | B42 | 71-79 / 77-85 / 83-91 | RPQVPLRPM / RPMTYKAAV /GAFDLSFFL | A / I |
|  | V85L |  | 0,0% | 0,6% |  | B42 | 77-85 / 83-91 | RPMTYKAAV /GAFDLSFFL | I |
|  | D86Y |  | 0,7% | 0,0% |  | B42 | 77-85 / 83-91 | RPMTYKAAV /GAFDLSFFL | A / I |
|  | L87I |  | 0,8% | 0,0% |  | B42 | 77-85 / 83-91 | RPMTYKAAV /GAFDLSFFL | A / I |
|  | H89N |  | 3,3% | 0,0% |  | B44 | 83-91 / 92-100 | GAFDLSFFL / KEKGGLEGL | I / A |
|  | F90L |  | 0,6% | 0,0% |  | B44 | 83-91 / 92-100 | GAFDLSFFL / KEKGGLEGL | I / A |
|  | G95W |  | 0,5% | 0,0% |  | B44 | 92-100 | KEKGGLEGL | I |
|  | G96V |  | 1,6% | 0,0% |  | B44 | 92-100 | KEKGGLEGL | I |
|  | G99W |  | 0,6% | 0,0% |  | B44 | 92-100 | KEKGGLEGL | I |
|  | G99V |  | 0,5% | 0,0% |  | B44 | 92-100 | KEKGGLEGL | I |
|  | I101V |  | 5,2% | 96,8% |  | B44 | 92-100 | KEKGGLEGL | A |
|  | H102Y |  | 4,9% | 97,1% |  | B44 | 92-100 / 105-115 | KEKGGLEGL / KRQEILDLWVY | A |
|  | H102N |  | 2,0% | 0,0% |  | B44 | 92-100 / 105-115 | KEKGGLEGL / KRQEILDLWVY | A |
|  | S103P |  | 0,5% | 0,0% |  | B44 | 92-100 / 105-115 | KEKGGLEGL / KRQEILDLWVY | A |
|  | S103Y |  | 1,0% | 0,0% |  | B44 | 92-100 / 105-115 | KEKGGLEGL / KRQEILDLWVY | A |
|  | Q104N |  | 0,9% | 0,0% |  | B44 | 105-115 | KRQEILDLWVY | A |
|  | R106G |  | 0,6% | 0,0% |  | B44 | 105-115 | KRQEILDLWVY | I |
|  | R106I |  | 1,0% | 0,0% |  | B44 | 105-115 | KRQEILDLWVY | I |
|  | D108G |  | 0,0% | 0,6% |  | B44 | 105-115 | KRQEILDLWVY | I |
|  | L110I |  | 0,6% | 0,0% |  | B44 | 105-115 | KRQEILDLWVY | I |
|  | L110P |  | 2,7% | 0,0% |  | B44 | 105-115 | KRQEILDLWVY | I |
|  | D111Y |  | 1,7% | 0,0% |  | B44 | 105-115 | KRQEILDLWVY | I |
|  | L112M |  | 0,9% | 0,0% |  | B44 | 105-115 | KRQEILDLWVY | I |
|  | W113R |  | 0,0% | 0,6% |  | B44 | 105-115 | KRQEILDLWVY | I |
|  | W113L |  | 0,6% | 0,0% |  | B44 | 105-115 | KRQEILDLWVY | I |
|  | I114A |  | 0,8% | 0,0% |  | B44 | 105-115 | KRQEILDLWVY | I |
|  | H116N |  | 94,5% | 2,4% |  | B44 | 105-115 | KRQEILDLWVY | I |
|  | Q125H |  | 1,4% | 0,0% |  | B42 | 128-137 | TPGPGVRYPL | A |
|  | P129Q |  | 5,7% | 96,5% |  | B42 | 128-137 | TPGPGVRYPL | I |
|  | V133I |  | 5,2% | 96,5% |  | B42 | 128-137 | TPGPGVRYPL | I |
|  | P136Q |  | 0,6% | 0,0% |  | B42 | 128-137 | TPGPGVRYPL | I |
|  | T138R |  | 0,8% | 0,0% |  | B42 | 128-137 | TPGPGVRYPL | A |
|  | G140V |  | 0,5% | 0,0% |  | B42 | 128-137 | TPGPGVRYPL | A |
| VC15 | M79I |  | 0,0% | 0,5% |  | B57 | 82-90 | KAAFDLSFF | A |
|  | V85L |  | 66,8% | 99,5% |  | B57 | 82-90 | KAAFDLSFF | I |
|  | E93G |  | 0,0% | 0,5% |  | B57 | 82-90 / 90-97 | KAAFDLSFF / FLKEKGGL | A / I |
|  | G96R |  | 0,0% | 2,4% |  | B57 | 90-97 | FLKEKGGL | I |
|  | H102Y |  | 68,4% | 99,7% |  | B57 | 105-115 | KRQEILDLWVY | A |
|  | R106G |  | 0,5% | 0,6% |  | B57 | 105-115 | KRQEILDLWVY | I |
|  | H116D |  | 0,0% | 0,8% |  | B57 | 105-115 / 116-124 | KRQEILDLWVY / HTQGYFPDW | A / I |
|  | Q125R |  | 0,0% | 1,7% |  | B57 | 116-124 / 120-128 / 127-135 | HTQGYFPDW / YFPDWQNYT / YTPGPGIRY | A / I |
|  | Y127C |  | 0,0% | 0,5% |  | B57 | 116-124 / 120-128 / 127-135 | HTQGYFPDW / YFPDWQNYT / YTPGPGIRY | A / I |
| VC16 | Y81F |  | 54,3% | 47,6% |  | B57 | 82-90 | KAAFDLSFF | A |
|  | H89Y |  | 0,0% | 29,3% |  | B57 | 82-90 / 90-97 | KAAFDLSFF / FLKEKGGL | I /A |
|  | K92R |  | 19,0% | 0,0% |  | B57 | 82-90 / 90-97 | KAAFDLSFF / FLKEKGGL | A / I |
|  | L97P |  | 0,6% | 0,0% |  | B57 | 90-97 | FLKEKGGL | I |
|  | H102Y |  | 99,9% | 48,6% |  | B14 / B57 | 105-113 / 105-115 | QRQDILDLW / KRQEILDLWVY | A |
|  | D111G |  | 1,9% | 0,0% |  | B14 / B57 | 105-113 / 105-115 | QRQDILDLW / KRQEILDLWVY | I |
|  | H116N |  | 63,4% | 63,1% |  | B57 | 116-124 | HTQGYFPDW | I |
|  | T117A |  | 0,6% | 0,0% |  | B57 | 116-124 / 120-128 | HTQGYFPDW / YFPDWQNYT | I / A |
|  | F121L |  | 0,5% | 0,0% |  | B57 | 116-124 / 120-128 | HTQGYFPDW / YFPDWQNYT | I |
|  | V133T |  | 99,4% | 13,3% |  | B57 | 127-135 | YTPGPGIRY | I |
|  | V133I |  | 0,0% | 74,3% |  | B57 | 127-135 | YTPGPGIRY | I |
|  | Y135H |  | 14,0% | 0,0% |  | B57 | 127-135 / 137-145 | YTPGPGIRY / LTFGWCFKL | I / A |
|  | Y143L |  | 0,0% | 0,6% |  | B57 | 137-145 | LTFGWCFKL | I |
|  | W183R |  | 1,0% | 0,0% |  | B14 | 183-191 | WRFDSRLAF | I |
|  | F185S |  | 1,9% | 0,0% |  | B14 | 183-191 | WRFDSRLAF | I |
|  | L189Q |  | 1,1% | 0,0% |  | B14 | 183-191 | WRFDSRLAF | I |
|  | F191H |  | 1,0% | 0,0% |  | B14 | 183-191 | WRFDSRLAF | I |
|  | F191Y |  | 0,7% | 0,0% |  | B14 | 183-191 | WRFDSRLAF | I |
|  | F191S |  | 1,2% | 0,0% |  | B14 | 183-191 | WRFDSRLAF | I |
|  | H192P |  | 3,2% | 0,0% |  | B14 | 183-191 | WRFDSRLAF | A |
|  | H193E |  | 2,9% | 0,0% |  | B14 | 183-191 | WRFDSRLAF | A |
|  | H193P |  | 0,0% | 0,8% |  | B14 | 183-191 | WRFDSRLAF | A |
|  | H193R |  | 0,0% | 0,6% |  | B14 | 183-191 | WRFDSRLAF | A |
|  | H193Q |  | 0,7% | 0,0% |  | B14 | 183-191 | WRFDSRLAF | A |
|  | V194Y |  | 6,5% | 0,0% |  | B14 | 183-191 | WRFDSRLAF | A |
|  | V194E |  | 0,0% | 0,9% |  | B14 | 183-191 | WRFDSRLAF | A |

* Consensus amino acid from available bulk sequence available showed shown instead of frequency; Location A - Adjacent to Epitope; Location I - Within Epitope
